# A large-scale RNA interference screen identifies genes that regulate autophagy at different stages

**DOI:** 10.1038/s41598-018-21106-5

**Published:** 2018-02-12

**Authors:** Sujuan Guo, Kevin J. Pridham, Ching-Man Virbasius, Bin He, Liqing Zhang, Hanne Varmark, Michael R. Green, Zhi Sheng

**Affiliations:** 1 Virginia Tech Carilion Research Institute, Roanoke, VA 24016 United States; 2 Graduate Program in Translational Biology, Medicine, and Health, Virginia Tech, Blacksburg, VA 24061 United States; 3 Howard Hughes Medical Institute and Department of Molecular, Cell and Cancer Biology, University of Massachusetts Medical School, Worcester, MA 01605 United States; 4 Department of Computer Science, Virginia Tech, Blacksburg, VA 24061 United States; 5 The Novo Nordisk Foundation Center for Basic Metabolic Research, University of Copenhagen, Copenhagen, Denmark; 6 Department of Biological Sciences and Pathobiology, Virginia-Maryland College of Veterinary Medicine, Virginia Tech, Blacksburg, VA 24061 United States; 7 Department of Internal Medicine, Virginia Tech Carilion School of Medicine, Roanoke, VA 24016 United States; 8 Faculty of Health Science, Virginia Tech, Blacksburg, VA 24061 United States

**Keywords:** Chronic myeloid leukaemia, Macroautophagy

## Abstract

Dysregulated autophagy is central to the pathogenesis and therapeutic development of cancer. However, how autophagy is regulated in cancer is not well understood and genes that modulate cancer autophagy are not fully defined. To gain more insights into autophagy regulation in cancer, we performed a large-scale RNA interference screen in K562 human chronic myeloid leukemia cells using monodansylcadaverine staining, an autophagy-detecting approach equivalent to immunoblotting of the autophagy marker LC3B or fluorescence microscopy of GFP-LC3B. By coupling monodansylcadaverine staining with fluorescence-activated cell sorting, we successfully isolated autophagic K562 cells where we identified 336 short hairpin RNAs. After candidate validation using Cyto-ID fluorescence spectrophotometry, LC3B immunoblotting, and quantitative RT-PCR, 82 genes were identified as autophagy-regulating genes. 20 genes have been reported previously and the remaining 62 candidates are novel autophagy mediators. Bioinformatic analyses revealed that most candidate genes were involved in molecular pathways regulating autophagy, rather than directly participating in the autophagy process. Further autophagy flux assays revealed that 57 autophagy-regulating genes suppressed autophagy initiation, whereas 21 candidates promoted autophagy maturation. Our RNA interference screen identified genes that regulate autophagy at different stages, which helps decode autophagy regulation in cancer and offers novel avenues to develop autophagy-related therapies for cancer.

## Introduction

Autophagy (also known as macroautophagy) is a subcellular catabolic process where cytoplasm and organelles are digested, and metabolic products are recycled to prolong cell survival under conditions of stress. Autophagy is characterized by the formation of autophagosomes (autophagy initiation) followed by fusion with lysosomes. The resulting autolysosomes degrade and recycle engulfed materials (autophagy maturation). This entire dynamic process is termed as autophagy flux. While autophagy often protects cells, excessive digestion triggers autophagic cell death. Hence, autophagy is critical for maintaining homeostasis and is central to the pathogenesis of many human diseases including cancer^1–4^.

Mounting evidence has established a fundamental role of autophagy in tumor initiation and progression^5^. As such, targeting autophagy as a means for cancer therapeutic development has been widely explored^6–9^. However, the relationship between autophagy and cancer therapeutic responses is complicated. On one hand, autophagy activators are appealing cancer drugs because excessive autophagy induces autophagic cell death. Many cancer signaling pathways such as PI3K/MTOR (phosphatidylinositol-4,5-bisphosphate 3-kinase/mechanistic target of rapamycin)^10–13^ or RAS (rat sarcoma viral oncogene homolog)^14,15^ suppress autophagy. However, targeting these kinases or small GTPase to specifically activate autophagic cell death has been proven difficult because of their important roles in other cell death pathways such as apoptosis^6,16,17^. On the other hand, established cancer often maintains a proficient level of autophagy to sustain cancer cell survival^18^, making autophagy blockade an attractive strategy to break this protection. Inhibition of autophagy can be achieved either by blocking autophagy initiation characterized by formation of autophagosomes or by impairing autophagy maturation to halt autophagy flux. Targeting autophagy maturation to block autophagy flux has been used in treating cancer. For example, chloroquine blocks the activity of autolysosomes to impede autophagy flux^19^. In combination with conventional therapies, this drug has prolonged the survival of glioblastoma patients; however, the statistical significance was low^20^. In addition, chloroquine exhibits severe side effects in the clinic limiting its application as an effective cancer treatment^21,22^. Many efforts have been invested to find new strategies to tackle autophagy with limited success, largely because autophagy regulation in cancer is not well understood and genes that regulate this dynamic process are not fully defined.

Chronic myeloid leukemia (CML) is characterized by the production of a single oncogenic protein BCR-ABL, which results from the translocation between chromosome 9 and 22^23^. Imatinib and its derivatives, the specific inhibitors of BCR-ABL, have been used in the clinic to treat CML^24^; however, resistance that is either BCR-ABL-dependent (i.e. BCR-ABL mutations) or -independent still develops in patients^25^. One possible reason for BCR-ABL-independent imatinib resistance is that CML cells activate autophagy to sustain their survival during imatinib treatment^26–29^. In our previous study, we show that BCR-ABL suppresses autophagy through activating a transcription factor called activating transcription factor 5 (ATF5), which in turn transcriptionally activates the expression of MTOR. Blocking ATF5 sensitizes CML cells to imatinib^26,28^. Based upon these results, it is imperative to identify novel autophagy regulators in CML so as to not only gain deeper insights into autophagy regulation but also provide novel therapeutic targets to develop effective autophagy-related therapies for cancer.

In this report, we isolated autophagic K562 cells using the fluorescent dye, monodansylcadaverine (MDC), coupled with fluorescence-activated cell sorting (FACS). The sensitivity of this approach in detecting autophagy was similar to other autophagy-detecting approaches such as LC3B immunoblotting and Cyto-ID fluorescence spectrophotometry. We carried out a large-scale RNA interference (RNAi) screen followed by rigorous multistep validations. We identified 82 genes that regulate autophagy in K562 cells. Among these genes, 62 candidates are novel autophagy regulators. Furthermore, we revealed that these genes either suppress autophagy initiation or promote autophagy maturation. Our results have important implications in understanding fundamental mechanisms of autophagy regulation in cancer and in the development of effective cancer therapies.

## Results

### MDC stains autophagic K562 cells

**Figure 1:**
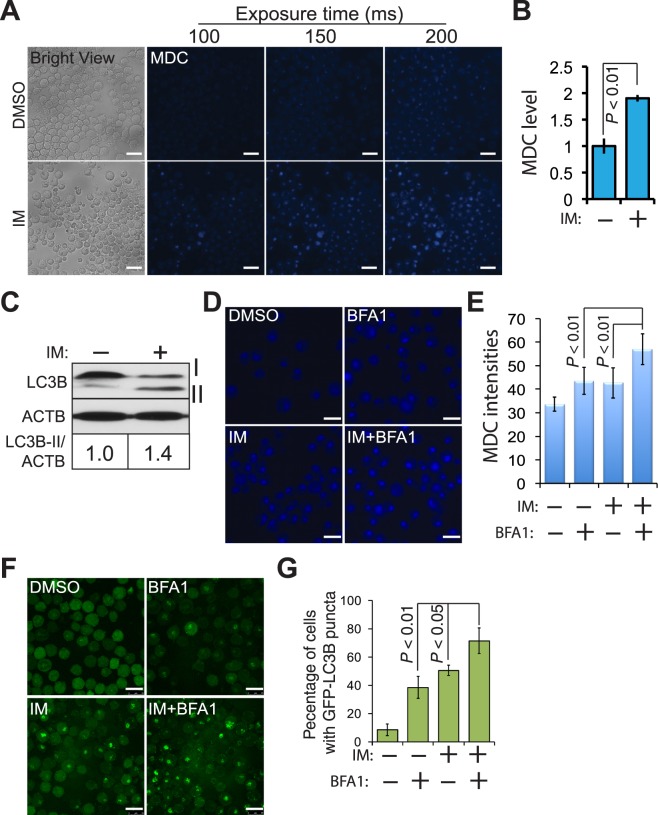
Monodansylcadaverine (MDC) stains autophagic K562 cells with sensitivity equivalent to LC3B immunoblotting and GFP-LC3B fluorescence microscopy. (**A**) Fluorescence microscopy. K562 cells were treated with dimethyl sulfoxide (DMSO) or 1 μM imatinib (IM) overnight followed by MDC staining. Images of live cells were taken at different exposure times (milliseconds, ms) using an inverted fluorescence microscope. Scale bar: 25 μm. (**B**) MDC Fluorescence spectrophotometry. K562 cells treated with DMSO or IM were stained with MDC. MDC fluorescence of live K562 cells was quantified using a micro-plate reader at an excitation wavelength of 335 nm and an emission wavelength of 525 nm. The relative MDC levels were obtained by dividing MDC fluorescence of IM-treated cells to that of DMSO-treated cells. Error bars represent three independent experiments. (**C**) LC3B immunoblotting. Cropped images are shown and full images are included in supplemental materials. ACTB (β actin) is the loading control. The intensities of LC3B-II or ACTB were quantified using Image J. The fold changes of LC3B-II/ACTB were obtained by dividing the ratio of LC3B-II/ACTB in IM-treated cells with that of DMSO-treated cells. (**D**) MDC staining of K562 cells treated with IM and/or bafilomycin A1 (BFA1). K562 cells were treated with a combination of 1 μM IM (10 hours) and 5 nM of BFA1 (48 hours). Cells were imaged using a fluorescence confocal microscope. (**E**) Quantification of MDC intensities. Fluorescence intensities of MDC in 10 different cells from three images were quantified using Image J. (**F**) GFP-LC3B fluorescence microscopy. K562 cells were stably transfected with a construct encoding GFP-LC3B. Cells were treated with a combination of IM and BFA1 and then imaged. (**G**) Quantification of cells with GFP-LC3B puncta. Four to five images of more than thirty K562 cells from each treatment were randomly selected for the following quantification analyses. GFP-expressing K562 cells or K562 cells with GFP puncta (indicating autophagy) were counted by three persons. Percentages of K562 cells with puncta were obtained by dividing numbers of K562 cells with GFP puncta with those of GFP-expressing cells. Scale bar: 25 nm.

Labeling and isolating live autophagic cells is difficult^30^. To resolve this issue, we employed a fluorescent dye MDC that labels acidic compartments including autolysosomes^31^. To stain live autophagic cells with MDC, we treated K562 cells with 1 μM imatinib (IM) to induce autophagy in K562 cells as described in our previous report^26^. We then stained K562 cells with MDC and monitored MDC fluorescence in live cells using an inverted fluorescence microscope. MDC fluorescence was in low levels and barely detected in live K562 cells treated with DMSO (top panel), whereas strong MDC fluorescence was detected in IM-treated cells (Fig. 1A, bottom panel). To corroborate these results and quantitatively measure MDC fluorescence, we carried out a spectrophotometric assay using a microplate reader. IM significantly enhanced MDC fluorescence intensities by approximately 2-fold (Fig. 1B). LC3B (microtubule associated protein 1 light chain 3 β, MAP1LC3B) is an autophagy marker. Immuno-detection of the lipidated form of LC3B (LC3B-II) located on the membrane of autophagosomes and GFP-LC3B fluorescence microscopy that monitors the formation of autophagosomes (GFP-LC3B puncta) are well-established autophagy assays^32,33^. We determined levels of LC3B-II in K562 cells treated with DMSO or IM. IM induced a 1.4-fold increase of LC3B-II (Fig. 1C), similar to the fold changes of MDC fluorescence. Hence, the sensitivity of MDC in labeling autophagic cells is equivalent to that of LC3B. To determine whether MDC staining can be used to measure autophagy flux, we treated K562 cells with IM and bafilomycin A1 (BFA1), a selective inhibitor of vacuolar H^+^ ATPase that prevents the fusion of autophagosomes and lysosomes. Cells treated with both IM and BFA1 showed much stronger MDC fluorescence (Fig. 1D). Quantification revealed that the co-treatment of IM and BFA1 significantly enhanced MDC intensities compared to each treatment alone (Fig. 1E). To compare MDC staining with GFP-LC3B fluorescence microscopy, we transfected K562 cells with GFP-LC3B followed by the treatment of IM and/or BFA1. IM and BFA1 significantly increased percentages of cells with GFP-LC3B puncta (Fig. 1F,G), congruent with results of MDC staining (Fig. 1D,E). We did notice that the background levels of MDC staining were much higher than those of GFP-LC3B puncta. However, GFP-LC3B fluorescence microscopy is based on the morphological changes of GFP-LC3B rather than fluorescence intensity. As such, GFP-LC3B-based assays are not suitable for isolating autophagic cells using FACS. While MDC may stain cells with enhanced levels of both lysosomes and autolysosomes (see discussion for details), this approach is compatible with FACS and suitable for high-content studies. In addition, our results shown above demonstrate that MDC staining can be used to determine autophagy and autophagy flux at least in K562 cells upon IM treatment. We therefore used MDC staining coupled with FACS to isolate autophagic K562 cells in the following experiments.

### A genome-wide RNAi screen identifies novel autophagy-regulating genes

**Figure 2:**
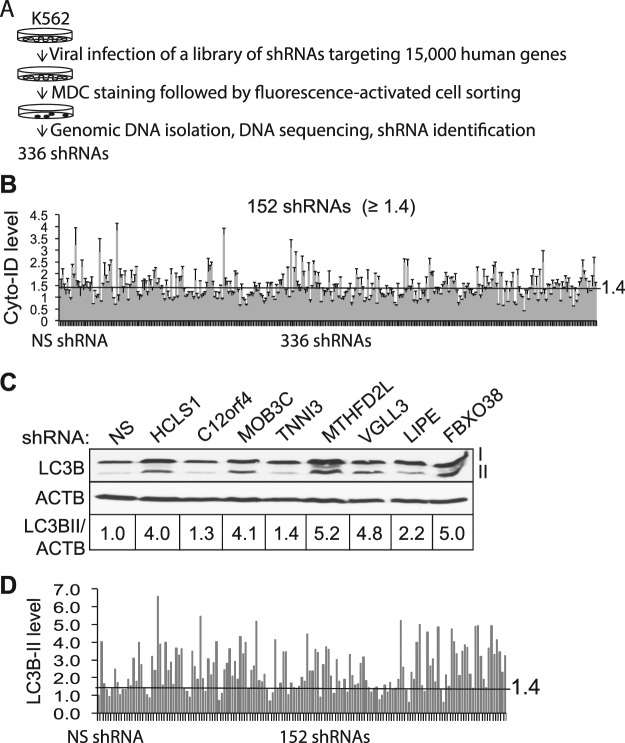
A large-scale RNA interference screen identifies autophagy-regulating genes. (**A**) Schematic diagram illustrating a large-scale RNA interference screen. 336 shRNAs were identified in MDC positive cells. (**B**) Candidate validation using the Cyto-ID spectrophotometric assay. K562 cells were treated with non-silencing (NS) shRNA or individual 336 candidate shRNAs. The 1.4-fold increase of Cyto-ID fluorescence was empirically set as the cut-off line. (**C**) Candidate validation using the LC3B immunoblotting assay. K562 cells were treated with NS or 152 individual shRNAs of Cyto-ID positive candidates. Autophagy was monitored using LC3B immunoblotting. A cropped representative blot of LC3B immunoblotting is shown. Full images are included in supplemental materials. (**D**) Quantification of LC3B-II intensities. The intensities of protein bands were quantified using Image J. The ratios of the protein intensities of LC3B-II and ACTB are shown. The 1.4-fold increase of LC3B-II was empirically set as the cut-off line.

Next, we employed MDC staining followed by FACS in a genome-wide RNAi screen designed for identifying novel autophagy regulators. As illustrated in Fig. 2A, K562 cells were transduced with a library of 75,000 shRNAs that target nearly 15,000 human genes. After isolation of autophagic cells using MDC and FACS, genomic DNAs that harbor shRNAs were extracted. shRNAs were PCR-amplified using pre-designed primers, and subsequently identified using Sanger DNA sequencing. In more than 500 individual PCR products, we identified 336 shRNAs (Fig. 2A and Table S1) and more than 50 shRNAs were repeatedly sequenced.

To further validate these candidates we employed another fluorescent dye, Cyto-ID, which specifically labels autophagosomes, amphisomes, and autolysosomes with minimal staining of lysosomes^34,35^. We transduced K562 cells with viruses containing individual shRNAs or a control non-silencing (NS) shRNA. Autophagy was measured using the Cyto-ID fluorescence spectrophotometric assay we have recently developed^34,35^. Among 336 candidates, 152 shRNAs induced a ≥ 1.4-fold increase of Cyto-ID fluorescence, compared to NS shRNA (Fig. 2B and Table S1). We next utilized LC3B immunoblotting to analyze 152 Cyto-ID positive candidates. A representative blot was shown in Fig. 2C. We found that 124 shRNAs increased LC3B-II intensities by ≥ 1.4-fold based on quantification of protein blots (Fig. 2D and Table S2). We empirically chose 1.4-fold increase of Cyto-ID fluorescence or LC3B-II as the cutoff lines because this approach allows us to include autophagy regulators with differential capabilities to activate autophagy (see below for details). The strength of autophagy responses often determines cell fate. For instance, autophagy often protects cells from cell death; however, a strong response often induces excessive autophagy leading to cell death.

**Figure 3:**
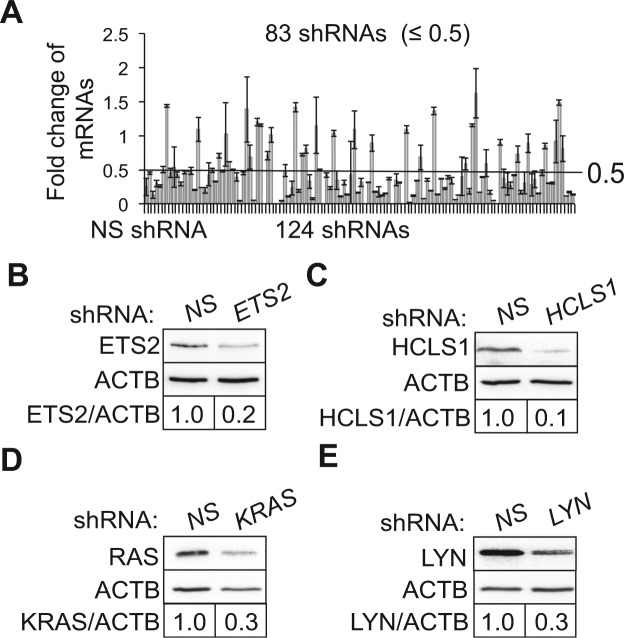
Knockdown efficiency of candidate autophagy-regulating genes (ARGs). (**A**) Quantitative RT-PCR. K562 cells were treated NS or 124 individual shRNAs of Cyto-ID and LC3B-II positive candidates. mRNA levels of shRNA-targeting genes were measured using quantitative RT-PCR. The cut-off line was set as 0.5. Error bars represent standard deviations from three independent experiments. Protein levels of ETS2 (**B**), HCLS1 (**G**), KRAS (**D**), and LYN (**E**) in K562 cells treated with their shRNAs were determined using immunoblotting. Cropped images are shown and full images are included in supplemental materials. Protein levels were quantified using Image J. ACTB (β actin) is the loading control. Fold changes of ARG protein levels were obtained by dividing the ratios of ARG/ACTB in ARG shRNA-treated cells to those in NS shRNA-treated cells.

To determine whether a specific shRNA is on or off target, we measured knockdown efficiency of 124 shRNAs on 122 target genes using quantitative RT-PCR. We found that 83 shRNAs induced a ≥2-fold reduction in mRNA levels of 82 genes (Fig. 3A and Table S3). We then selected four candidates and validated knockdown at protein levels using commercially available antibodies. shRNAs of *ETS2*, *HCLS1*, *KRAS* and *LYN* remarkably decreased protein expression at a factor of 2 to 5 folds (Fig. 3B–E), congruent with RT-PCR results.

82 candidate genes were hereafter dubbed autophagy-regulating genes (ARGs, Table 1). As mentioned in our previous report, *ATF5* regulates IM-induced autophagy in BCR-ABL positive CML cells^26^. The identification of *ATF5* as an ARG in K562 CML cells indicates that the RNAi screen described above is unbiased. In addition to *ATF5*, 19 ARGs (*ACAT1, CAMKV*, *CNOT2*, *ERGIC1*, *ETS2*, *HSF2*, *IGF2R*, *JAK1*, *KRAS*, *LPCAT2*, *LYN*, *PAH*, *PEX2*, *PLK1*, *PRKCD*, *S100A4*, *SCD*, *VAMP7*, and *YWHAZ*) were also reported previously as autophagy modulators^26,36–54^. Hence, the remaining 62 ARGs are novel autophagy mediators. The fact that approximately one-fourth of ARGs has been reported previously verifies the feasibility of MDC in isolating autophagic cells and the rigor of multistep validations in this large-scale RNAi screen. It is also notable that none of the 82 ARGs are autophagy-related genes, which are structural proteins or enzymes that directly participate in the formation of autophagic compartments such as autophagosomes and autolysosome^55^. This is perhaps because knockdown of autophagy-related genes impairs the formation of autophagic compartments and, therefore, is expected to decrease–rather than increase–the levels of MDC, Cyto-ID, or LC3B-II. Hence, a different approach needs to be taken to measure activities of autophagy-related genes during autophagy activation or inhibition in future RNA interference screens.

**Table 1:** Autophagy-regulating genes.

Gene Symbol	Human Gene Descriptions	Gene Symbol	Human Gene Descriptions
ACAT1*	acetyl-Coenzyme A acetyltransferase 1	LPCAT2*	lysophosphatidylcholine acyltransferase 2
*ADAMTS2*	ADAM metallopeptidase with thrombospondin type 1 motif, 2	LYN*	v-yes-1 Yamaguchi sarcoma viral related oncogene homolog
*AGPS*	alkylglycerone phosphate synthase	*MBTPS1*	membrane-bound transcription factor peptidase, site 1
*AK9*	adenylate kinase 9	*MESP2*	mesoderm posterior 2 homolog (mouse)
*AKIP1*	A kinase (PRKA) interacting protein 1	*MOB3C*	MOB Kinase Activator 3C
*AKR1C3*	aldo-keto reductase family 1, member C3	*NCS1*	Neuronal Calcium Sensor 1
ATF5*	activating transcription factor 5	*NSDHL*	NAD(P) dependent steroid dehydrogenase-like
*BVES*	blood vessel epicardial substance	PAH*	phenylalanine hydroxylase
*C1QL3*	complement component 1, q subcomponent-like 3	PEX2*	Peroxisomal Biogenesis Factor 2
CAMKV*	CaM kinase-like vesicle-associated	*PGLYRP3*	peptidoglycan recognition protein 3
*CBWD1*	COBW domain containing 1	*PHF14*	PHD finger protein 14
*CCDC108*	coiled-coil domain containing 108	*PIPOX*	pipecolic acid oxidase
*CCDC77*	coiled-coil domain containing 77	PLK1*	polo-like kinase 1 (Drosophila)
*CENPH*	centromere protein H	*POLR3D*	polymerase (RNA) III (DNA directed) polypeptide D, 44 kDa
*CLECL1*	C-type lectin-like 1	*PPP3R2*	protein phosphatase 3 (formerly 2B), regulatory subunit B, beta isoform
*CLTA*	clathrin, light chain (Lca)	PRKCD*	protein kinase C, delta
CNOT2*	CCR4-NOT transcription complex, subunit 2	*PTDSS1*	phosphatidylserine synthase 1
*COX11*	COX11 homolog, cytochrome c oxidase assembly protein (yeast)	*RNASEH1*	ribonuclease H1
*CPXM1*	carboxypeptidase X (M14 family), member 1	*RP9*	retinitis pigmentosa 9 (autosomal dominant)
*CUBN*	cubilin (intrinsic factor-cobalamin receptor)	S100A4*	S100 calcium binding protein A4
*DIRC1*	disrupted in renal carcinoma 1	*SASH3*	SAM and SH3 domain containing 3
*DNASE1L1*	deoxyribonuclease I-like 1	SCD*	stearoyl-CoA desaturase (delta-9-desaturase)
ERGIC1*	endoplasmic reticulum-golgi intermediate compartment (ERGIC) 1	*SEPT1*	septin 1
*ERLIN2*	ER lipid raft associated 2	*SERP1*	stress-associated endoplasmic reticulum protein 1
ETS2*	v-ets erythroblastosis virus E26 oncogene homolog 2 (avian)	*SLC25A33*	solute carrier family 25, member 33
*FABP6*	fatty acid binding protein 6, ileal	*SLC2A8*	solute carrier family 2 (facilitated glucose transporter), member 8
*FAM13C1*	family with sequence similarity 13, member C1	*TBX15*	T-box 15
*FBXO38*	F-box protein 38	*TCERG1*	transcription elongation regulator 1
*GGCT*	gamma-glutamyl cyclotransferase	*THAP2*	THAP domain containing, apoptosis associated protein 2
*GH2*	growth hormone 2	*TNNI3*	troponin I type 3 (cardiac)
*GLO1*	glyoxalase I	*U2SURP*	U2 SnRNP-Associated SURP Domain Containing
*GPR19*	G protein-coupled receptor 19	*UBA6*	ubiquitin-like modifier activating enzyme 6
*GRHL1*	grainyhead-like 1 (Drosophila)	*UTP15*	UTP15, U3 small nucleolar ribonucleoprotein, homolog (S. cerevisiae)
*GRK4*	G protein-coupled receptor kinase 4	VAMP7*	vesicle-associated membrane protein 7
*HCLS1*	hematopoietic cell-specific Lyn substrate 1	*VGLL3*	vestigial like 3 (Drosophila)
HSF2*	heat shock transcription factor 2	*WBP1L*	WW domain binding protein 1-like
IGF2R*	insulin-like growth factor 2 receptor	YWHAZ*	tyrosine 3-monooxygenase/tryptophan 5-monooxygenase activation protein, zeta polypeptide
*IGSF1*	immunoglobulin superfamily, member 1	*ZFP1*	zinc finger protein 1 homolog (mouse)
JAK1*	Janus kinase 1 (a protein tyrosine kinase)	*ZNF197*	zinc finger protein 197
KRAS*	v-Ki-ras2 Kirsten rat sarcoma viral oncogene homolog	*ZNF330*	zinc finger protein 330
*LINC00467*	long intergenic non-protein coding RNA 467	*ZNF639*	zinc finger protein 639

*ARGs previously reported as autophagy mediators.

Cyto-ID labels most autophagic compartments such as autophagosomes, amphisomes, and autolysosomes^34^, whereas LC3B-II mostly highlights autophagosomes and autolysosomes^32,33^. Based on the changes in Cyto-ID fluorescence (Fig. 2B and Table S1) and LC3B-II intensities (Fig. 2D and Table S2), we defined the ability of ARG shRNAs to induce the formation of autophagic compartments using the following criteria: (1) High, if there was a ≥ 2-fold increase in both Cyto-ID and LC3B-II; (2) Medium, if there was a ≥ 2-fold increase of either Cyto-ID or LC3B-II; (3) Low, if there was a 1.4- to 1.9-fold increase of Cyto-ID and/or LC3B-II. More than half of ARG shRNAs was at medium levels, whereas 14 or 22 ARG shRNAs were at high or low levels, respectively (Table S4). Hence, ARGs have differential capabilities to modulate autophagy.

Taken together, our genome-wide RNAi screen followed by comprehensive and rigorous validations, identifies 82 ARGs with different capabilities in regulating autophagy in K562 cells. Of note, 62 candidate ARGs are novel autophagy mediators.

### Bioinformatic analyses of ARGs

**Figure 4:**
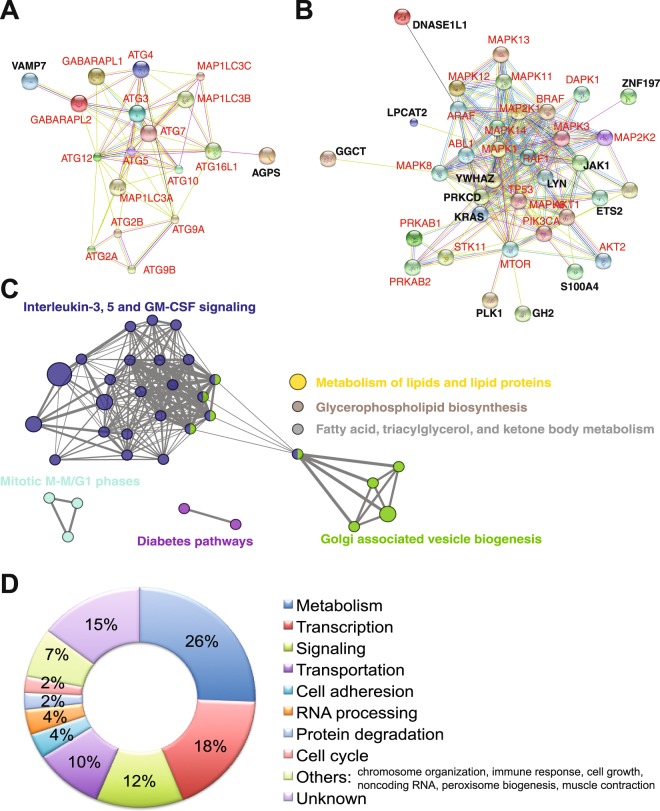
Bioinformatic analyses. (**A**) Predicted protein-protein interactions between candidate genes and core autophagy-related genes using the STRING program. 16 core autophagy-related genes (in red) were used as bait in the STRING database. ARGs (black) that show potential interactions with the bait are shown. Each line represents a potential interaction from one assay. The size of nodes represents the protein size. (**B**) Potential interactions between candidate genes and kinases in autophagy signaling. (**C**) Gene term enrichment analysis using the DAVID program. The biological processes that were significantly enriched were listed and color-coded. Each node represents genes involved in these biological processes. The larger the node, the more genes it represents. Two nodes are connected if they share genes or have possible interactions. (**D**) Biological processes in which candidate genes are involved. By using the data from the GeneCards database, candidate genes were categorized into different biological processes. The percentages of genes involved in these processes are shown.

To further characterize ARGs, we performed several bioinformatic analyses. We queried the STRING v9.1 database^56^, which predicts protein-protein interactions. By using 16 core autophagy proteins (e.g. ATG3, ATG4, and ATG5, etc.) and LC3B (MAP1LC3B) as the bait (Fig. 4A, in red), we found that only two ARG proteins VAMP7 and AGPS (Fig. 4A, in black) showed potential interactions with the bait proteins. In line with the result that no autophagy-related genes were found in our RNAi screen, these results suggest that ARGs identified herein do not directly participate in the process of autophagy. We then investigated whether ARG proteins interact with autophagy signaling molecules. We set 22 known autophagy signaling molecules (Fig. 4B, in red) as the bait and detected potential protein-protein interactions in 13 candidate ARG proteins (Fig. 4B, in black). Because 62 ARGs are novel autophagy mediators, there is perhaps a lack of information of protein-protein interactions in the database. Hence, these results suggest that ARGs are upstream mediators of autophagy process.

By using the DAVID program^57^, we performed gene ontology analyses. Most ARGs were directly or indirectly involved in the signaling pathways emanating from interleukin-3, interleukin-5, or granulocyte-macrophage colony-stimulating factor (Fig. 4C). These hematopoietic cytokines play vital roles in leukemogenesis, and are important for autophagy regulation in normal and malignant blood cells^58–60^. Only a few ARGs are implicated in mitosis, vesicle biogenesis, diabetes, and lipid metabolism. By querying the GeneCards database, we grouped ARGs based on the molecular pathways to which they possibly target. Percentages of ARGs that exhibit potential activities in metabolism, transcription, signaling, and molecule transportation were 26%, 18%, 12%, and 10%, respectively (Fig. 4D). These molecular pathways have crucial assignments in autophagy regulation^55,61,62^. These results have implications in understanding the relationship between autophagy and ARGs-targeting biological processes.

### ARGs target different stages of autophagy

Autophagy is a dynamic process (referred to as autophagy flux) including initiation (autophagosome formation) and maturation (autolysosome formation and cargo degradation). It is therefore crucial to distinguish whether the changes of autophagic compartments (autophagosomes, amphisomes, and autolysosomes) are due to autophagy activation or autophagy flux blockade because levels of autophagic compartments increase in both conditions^32,33^. In our RNAi screen and subsequent analyses, it is unclear whether the increase of MDC, Cyto-ID, and LC3B-II levels upon depletion of ARGs is due to the activation of autophagy initiation or the blockade of autophagy maturation.

**Figure 5:**
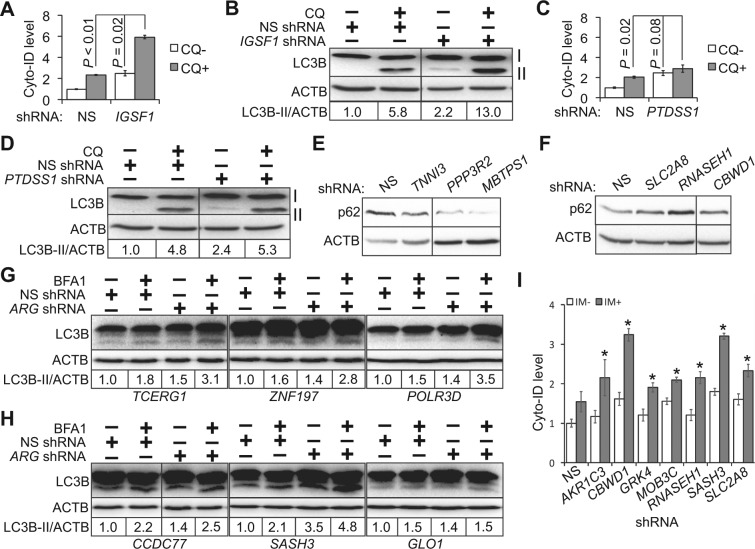
Determination of autophagy stages to which ARGs target. (**A**,**B**) Combination of *IGSF1* shRNA and chloroquine (CQ). K562 cells were treated with NS or *IGSF1* shRNA followed by chloroquine treatment. Autophagy was assessed using the Cyto-ID spectrophotometric assay (**A**) or LC3B immunoblotting (**B**). (**C**,**D**) Combination of PTDSS1 shRNA and CQ. K562 cells were treated with NS or *PTDSS1* shRNA. Autophagy was assessed using the Cyto-ID spectrophotometric assay (**C**) or LC3B immunoblotting (**D**). (**E**,**F**) p62 immunoblotting. K562 cells treated with NS shRNA or ARG shRNAs were subject to p62 immunoblotting. (**G**) Co-treatment of BFA1 and shRNAs of ARGs that suppress autophagy initiation. K562 cells were transduced with viruses harboring NS shRNA or shRNAs of *TCERG1*, *ZNF197*, or *POLR3D*. Cells were then treated with 5 nM of BFA1 for 48 h. Autophagy was monitored using LC3B immunoblotting. (**H**) Co-treatment of BFA1 and shRNAs of ARGs that promote autophagy maturation. (**I**). Autophagy flux in K562 cells treated with IM and/or shRNAs of ARGs that promote autophagy maturation. K562 cells were transduced with viruses containing NS shRNA or shRNAs of ARGs that promote autophagy maturation. Cells were then treated with DMSO (IM−) or 1 μM of IM (IM+). Autophagy was monitored using the Cyto-ID fluorescence spectrophotometric assay. ACTB is the loading control. Images cropped from same blot are shown and full images are included in supplemental materials. Error bars represent standard deviations of three independent experiments. *P < 0.05. *P* values determine the difference between combination of IM and ARG shRNA and each treatment alone.

To address this question, we employed the Cyto-ID fluorescence spectrophotometric assay. We have successfully monitored the changes of autophagic compartments in cells treated with autophagy activators and/or autophagy maturation blockers using this approach^34^. We treated ARG-deficient cells with chloroquine (an inhibitor of autophagy maturation)^29,63,64^. In principle, activation of autophagy in combination with chloroquine results in a significant increase of autophagy compartments compared to each treatment alone. For example, knockdown of *IGSF1* significantly increased Cyto-ID levels together with chloroquine (*P* < 0.05; Fig. 5A). Consistently, LC3B-II levels also substantially increased in cells treated with *IGSF1* shRNA and chloroquine (Fig. 5B). These results suggest that IGSF1 suppresses autophagy initiation and its depletion activates autophagy. In contrast, shRNAs of *PTDSS1* failed to induce a significant increase of Cyto-ID (*P* = 0.08; Fig. 5C) or LC3B-II (Fig. 5D) in combination with chloroquine, compared to *PTDSS1* shRNA alone. These results suggest that PTDSS1 targets the autophagy maturation stage. We did notice that the Cyto-ID assay detected similar changes in cells treated with either ARG shRNAs or chloroquine (Fig. 5A,C), whereas LC3B-II protein levels were much higher in chloroquine-treated cells than in ARG shRNAs-treated cells (Fig. 5B,D). This discrepancy may be due to the fact that Cyto-ID measures levels of most autophagic compartments and LC3B-II only labels autophagosomes. Based on *P* values that determine the statistical significance of difference between means of the combinational treatment (chloroquine and shRNA) and those of chloroquine or shRNA (Tables S5 and 2**)**, we found that 57 ARGs significantly enhanced the levels of autophagic compartments together with chloroquine, indicating that these ARGs suppress autophagy initiation. The remaining 25 ARG shRNAs failed to do so (Tables S5 and 2, in bold).

**Table 2:** Effect of ARG shRNAs and chloroquine on the formation of autophagic compartments.

Gene Symbols	*P* values		Gene Symbols	*P* values		Gene Symbols	*P* values	
	Combo vs shRNA	Combo vs CQ		Combo vs shRNA	Combo vs CQ		Combo vs shRNA	Combo vs CQ
*ACAT1*	<0.01	<0.01	***GGCT***	<0.01	0.09	*PPP3R2*	0.04	0.01
*ADAMTS2*	0.04	<0.01	*GH2*	<0.01	<0.01	*PRKCD*	0.01	0.04
*AGPS*	0.03	0.01	***GLO1***	0.01	0.15	***PTDSS1***	0.08	0.02
*AK9*	0.02	<0.01	*GPR19*	0.02	<0.01	***RNASEH1***	0.02	0.41
*AKIP1*	<0.01	<0.01	*GRHL1*	0.02	<0.01	*RP9*	0.01	<0.01
***AKR1C3***	0.08	<0.01	***GRK4***	0.07	0.02	*S100A4*	0.04	<0.01
*ATF5*	<0.01	<0.01	*HCLS1*	<0.01	0.02	***SASH3***	0.07	<0.01
*BVES*	0.01	0.03	***HSF2***	0.01	0.06	*SCD*	<0.01	<0.01
***C1QL3***	<0.01	0.42	*IGF2R*	<0.01	<0.01	*SEPT1*	0.04	<0.01
*CAMKV*	<0.01	<0.01	*IGSF1*	0.02	<0.01	*SERP1*	<0.01	<0.01
***CBWD1***	<0.01	0.12	*JAK1*	0.01	<0.01	*SLC25A33*	0.03	<0.01
*CCDC108*	<0.01	0.01	*KRAS*	0.02	<0.01	***SLC2A8***	0.13	0.02
***CCDC77***	0.11	<0.01	*LINC00467*	<0.01	<0.01	***TBX15***	0.05	0.21
*CENPH*	<0.01	<0.01	*LPCAT2*	<0.01	0.02	*TCERG1*	<0.01	0.02
*CLECL1*	<0.01	<0.01	*LYN*	<0.01	<0.01	*THAP2*	0.01	<0.01
*CLTA*	<0.01	<0.01	*MBTPS1*	0.01	<0.01	*TNNI3*	<0.01	<0.01
*CNOT2*	0.02	<0.01	*MESP2*	<0.01	<0.01	***U2SURP***	0.06	0.02
***COX11***	0.01	0.28	***MOB3C***	0.13	0.04	***UBA6***	0.06	<0.01
*CPXM1*	<0.01	<0.01	*NCS1*	<0.01	<0.01	*UTP15*	0.02	<0.01
*CUBN*	0.01	<0.01	*NSDHL*	<0.01	0.04	*VAMP7*	<0.01	<0.01
*DIRC1*	0.02	<0.01	*PAH*	<0.01	<0.01	*VGLL3*	0.01	<0.01
***DNASE1L1***	0.08	<0.01	*PEX2*	0.01	<0.01	*WBPL1*	0.02	<0.01
***ERGIC1***	0.06	<0.01	***PGLYRP3***	0.06	<0.01	***YWHAZ***	0.01	0.12
*ERLIN2*	0.01	<0.01	***PHF14***	0.02	0.14	***ZFP1***	0.16	<0.01
*ETS2*	0.02	<0.01	*PIPOX*	0.04	<0.01	*ZNF197*	<0.01	<0.01
*FABP6*	<0.01	<0.01	***PLK1***	0.07	<0.01	*ZNF330*	0.02	<0.01
***FAM13C1***	0.02	0.47	*POLR3D*	<0.01	<0.01	*ZNF639*	<0.01	<0.01
*FBXO38*	<0.01	<0.01						

Note: Combo indicates the combinational treatment of shRNA and chloroquine (CQ). *P* values of combo vs CQ or combo vs shRNA indicate whether the increase of Cyto-ID levels in cells treated with combo is significantly higher than Cyto-ID levels in cells treated with either CQ or shRNA. ARGs with a *P* value larger than 0.05 are highlighted in bold. Means and standard deviations of each treatment were shown in Table S5.

To further determine whether these 25 ARGs target autophagy maturation stage, we performed a combinational treatment of individual ARG shRNAs and PP242. PP242 is a compound that inhibits mechanistic target of rapamycin and launches autophagy^65^. In principle, knockdown of an ARG that promotes autophagy maturation will significantly increase the quantities of autophagic compartments together with PP242. We found that 21 ARG shRNAs exhibited statistical significance in enhancing the Cyto-ID fluorescence together with PP242 (highlighted in bold, Tables S6 and 3). As such, these ARGs facilitate the completion of the autophagy process through promoting autophagy maturation. To corroborate these results, we also monitored the changes of another autophagy marker p62 (sequestosome 1, SQSTM1), which is degraded in autolysosomes. Activation of autophagy often leads to decreased levels of p62, whereas blockade of autophagy maturation causes accumulation of this protein. We found that shRNAs of *TNNI3*, *PPP3R2*, or *MBTPS1* (ARGs that suppress autophagy initiation; Table 2) significantly reduced levels of p62 proteins (Fig. 5E). In contrast, p62 protein levels increased in cells treated with shRNAs of *SLC2A8*, *RNASEH1*, or *CBWD1* (Fig. 5F), which impaired autophagy maturation (Tables 2 and 3). To further validate our autophagy flux assay presented above, we used BFA1 to block autophagy flux (Fig. 1D–G) followed by LC3B immunoblotting. We found that depleting ARGs that target autophagy initiation (e.g. *TCERG1*, *ZNF197*, or *POLR3D*) led to a significant increase of LC3B-II levels upon BFA1 treatment (Fig. 5G), whereas knockdown of ARGs that target autophagy maturation (e.g. CCDC77, SASH3, or GLO1) failed to do so (Fig. 5H). Furthermore, an autophagy flux assay using a combination of imatinib (activating autophagy) and shRNAs of ARGs that promote autophagy maturation revealed that depletion of these ARGs significantly increased levels of autophagic compartments in combination with IM (Fig. 5I), consistent with PP242 treatment (Table 3).

**Table 3:** Effect of ARG shRNAs and PP242 on the formation of autophagic compartments.

Gene Symbol	*P* values	
	Combo vs PP242	Combo vs shRNA
***AKR1C3***	0.030	0.01
***C1QL3***	<0.01	<0.01
***CBWD1***	<0.01	<0.01
***CCDC77***	<0.01	<0.01
***COX11***	<0.01	<0.01
***DNASE1L1***	<0.01	<0.01
*ERGIC1*	0.425	<0.01
***FAM13C1***	0.01	<0.01
***GGCT***	0.047	<0.01
***GLO1***	<0.01	<0.01
***GRK4***	<0.01	<0.01
***HSF2***	0.024	<0.01
***MOB3C***	<0.01	<0.01
***PGLYRP3***	<0.01	<0.01
*PHF14*	0.096	<0.01
***PLK1***	0.025	<0.01
***PTDSS1***	<0.01	<0.01
***RNASEH1***	<0.01	<0.01
***SASH3***	<0.01	<0.01
***SLC2A8***	0.049	<0.01
*TBX15*	0.095	0.111
***U2SURP***	<0.01	<0.01
*UBA6*	0.266	<0.01
***YWHAZ***	<0.01	<0.01
***ZFP1***	<0.01	<0.01

Note: Combo indicates the combinational treatment of shRNA and PP242. *P* values of combo vs PP242 or combo vs shRNA indicate whether the increase of Cyto-ID levels in cells treated with combo (PP242 + shRNA) is significantly higher than Cyto-ID levels in cells treated with either PP242 or shRNA. ARGs with *P* values larger than 0.05 are not highlighted in bold. Means and standard deviations of each treatment were shown in Table S6.

Of note, shRNAs of four ARGs (*ERGIC1*, *PHF14*, *TBX15*, or *UBA6*) did not significantly change the levels of autophagic compartments together with either chloroquine (Table 2) or PP242 (Tables S6 and 3). One possible explanation is that these ARGs may inhibit PP242-induced autophagy, thereby diminishing a further increase of Cyto-ID fluorescence in cells upon combinational treatments. ARGs that target autophagy at different stages were summarized in Fig. S1. To determine the functional relevance of ARGs in CML, we measured the cytotoxicity of IM in K562 cells upon depletion of ARGs. We found that depletion of ARGs that promote autophagy maturation sensitized K562 cells to IM. In contrast, knockdown of ARGs that suppress autophagy initiation showed no additive or synergistic inhibition of cell viability (Fig. S2). These results not only provide a further validation of candidate ARGs in autophagy regulation, but also highlight the importance of ARGs as potential therapeutic targets in CML and other cancers.

## Discussion

In this study, we conducted a large-scale RNAi screen in K562 cells and identified 82 ARGs with 62 candidates as novel autophagy mediators. Our further analyses revealed two subclasses of ARGs: ARGs that suppress autophagy initiation and ARGs that promote autophagy maturation (Fig. S1). Identification of new autophagy regulators in cancer cells not only helps understand how autophagy is activated or inhibited, but also provides potential drug targets for future therapeutic intervention.

RNAi screens have been used previously in mammalian cells to identify genes that regulate autophagy. For instance, Chan *et al*., transiently transfected GFP-LC3B-positive HEK293 cells with small interfering (si) RNAs targeting human kinases and searched for kinases that block starvation-induced autophagy^66^. Following this study, Lipinsky *et al*.,^44^ and McKnight *et al*.,^67^ performed a genome-wide siRNA-based RNAi screen and identified genes important for autophagy activation during starvation. Several other research groups studied autophagy flux or autophagy signaling in unstressed conditions using siRNA or shRNA-based RNAi screening^68–71^. In these screens, LC3B immunoblotting or microscopic imaging of GFP-LC3B or GFP-p62 was used to detect autophagy. However, the workload of LC3B immunoblotting of individual shRNA-treated cells in a large-scale RNAi screen is overwhelming and time-consuming. Fluorescence imaging of GFP-LC3B or GFP-p62 depends on the availability of a high content and expensive imaging system. To date, only a few laboratories could afford to use this high-content imaging system in large-scale RNAi screens^44,67,69,70,72–74^.

In this report, we coupled MDC staining with FACS and successfully isolated autophagic K562 cells. Past research demonstrates that the specificity of MDC in labeling autophagic cells is relatively low compared to LC3B or p62 because this fluorescence dye non-specifically stains lysosomes^32,33^. However, our results shown in Figs 1, 2 indicate that MDC staining is at least feasible and efficient in labeling autophagic K562 cells. The sensitivity of this approach is equivalent to LC3B immunoblotting and GFP-LC3B fluorescence microscopy. While we could not rule out the possibility of non-specific staining of non-autophagic K562 cells by MDC in our primary screen, our rigorous validations using the Cyto-ID spectrophotometric assay and LC3B immunoblotting assay effectively eliminated the false positive candidates. This is supported by the fact that one-fourth of candidate ARGs have been reported previously. Hence, using MDC-based FACS to isolate autophagic cells is suitable for performing high-content screens (e.g. loss-of-function screens, gain-of-function screens, and small molecule screens, etc.) in normal or malignant cells. More importantly, this approach is inexpensive and affordable.

As described earlier, autophagy has multifaceted activities in cancer. On one hand, autophagy protects cancer cells from therapy-induced cell death. On the other hand excessive autophagy, by itself, induces cell death in cancer cells^18^. Hence, the determination of autophagy stages to which 82 ARGs target has important clinical implications in treating cancer. For example, 21 ARGs that target the autophagy maturation stage are of interest in developing novel combinational therapies in that effectively blocking autophagy maturation inhibits autophagy-promoted survival of CML cells, thereby enhancing the sensitivity of CML cells to BCR-ABL inhibitors such as IM^29,75^. Indeed, our results shown in Fig. S2 demonstrate the functional relevance of ARGs with IM sensitivity in K562 cells. Given the possibility of excessive autophagy in inducing cell death, 57 ARGs that suppress autophagy initiation are potential new therapeutic targets for CML. Our future research will uncover the therapeutic potential of 82 ARGs reported herein in CML or other cancers that benefit from autophagy-related therapies.

## Methods

### Materials and reagents

K562 cells were maintained in RPMI-1640 medium supplemented with 10% fetal bovine serum, 100 μg/ml streptomycin, and 100 μg/ml penicillin. The Cyto-ID kit was purchased from Enzo Life Sciences. PP242, chloroquine, bafilomycin A1 and monodansylcadaverine (MDC) were purchased from Sigma-Aldrich. Imatinib was purchased from LC Laboratories. Stock solutions were prepared as follows: imatinib (10 mM) was dissolved in either sterile water or DMSO; PP242 (20 mM) was dissolved in DMSO; chloroquine (10 mM) was dissolved in sterile water; bafilomycin A1 (5 mM) was dissolved in DMSO.

### Fluorescence microscopy

Fluorescence microscopy was used to monitor MDC fluorescence and GFP-LC3B. For MDC fluorescence microscopy, K562 cells were treated with a low dose of imatinib (1 μM) to induce autophagy^26^ or 5 nM of bafilomycin A1 to block autophagy flux. Cells were then stained with 0.1 mM MDC for 10 minutes in the dark at 37 °C as described previously^31^. Live MDC positive cells were imaged using an inverted fluorescence microscope (Carl Zeiss Microscopy, LLC, Thornwood, NY). Photos were taken using a 20× or 40× lens at different exposure times (from 100 to 200 milliseconds). For GFP-LC3B microscopy, K562 cells were stably transfected with a pEGFP-LC3B plasmid that encodes a fusion protein GFP-LC3B. Cells were then treated with imatinib (1 μM) for 18 hours and/or BFA1 (5 nM) for 48 hours. GFP fluorescence images were taken using a TCS SP8 laser scanning confocal microscope equipped with a Plan Apochromat 63×/1.4 numerical aperture oil immersion objective, and a Leica HyD hybrid detector (Leica, Buffalo Grove, IL).

### Quantification of cells with GFP-LC3B puncta

Four to five images of more than 30 K562 cells treated with DMSO, 1 μM imatinib, and/or 5 nM bafilomycin A1 were randomly selected. GFP-LC3B-expressing K562 cells or K562 cells with GFP-LC3B puncta (indicating the formation of autophagosomes) in these images were counted by three persons. Percentages of cells with GFP-LC3B puncta were obtained by dividing numbers of cells with GFP-LC3B puncta with those of GFP-LC3B-expressing cells. The statistical difference was obtained using the Student’s *t* test.

### Fluorescence spectrophotometric assay

The MDC or Cyto-ID fluorescence spectrophotometric assay was previously described^34,35^. In brief, autophagy was induced or inhibited in K562 cells as described in each figure. To minimize spontaneous autophagy due to lack of nutrients, cells were seeded in freshly prepared media at 5 × 10^4^ or 10^5^ cells per ml overnight. Live autophagic cells were stained with MDC as described above or with Cyto-ID based on the manufacturer’s instructions (Enzo Life Sciences, Inc.). Cells were then seeded in a 96-well plate and fluorescence intensity was quantified using a microplate reader (Molecular Devices, Sunnyvale, CA). The wavelengths used for detecting MDC fluorescence were 335 nm (excitation) and 525 nm (emission), respectively. The Cyto-ID fluorescence was measured at excitation 485 nm and emission 535 nm. Fluorescence levels were shown as fold changes by normalizing fluorescence intensities in cells treated with bafilomycin A1, imatinib, or shRNAs to those in untreated or non-silencing (NS) shRNA-treated cells. There were three biological replicates in each treatment group. The statistical difference was obtained using the Student’s *t* test.

### Lentivirus production

shRNAs used in this study were built on the pLKO.1 lentiviral vector. To produce lentivirus that harbors a library of about 75,000 shRNAs targeting 15,000 human genes (OpenBiosystem, GE Dharmacon), shRNA plasmids were divided into 16 pools and used to transfect HEK293T cells using effectene (QIAGEN). 2 μg of plasmid DNA was used to transfect 5 × 10^6^ cells to yield 3 ml media containing lentivirus. A series of dilution of viral supernatant was then used to transduce HEK293T cells and puromycin (0.25 μg/ml) was used to select infected cells. Based on cell colonies stained by crystal violet (Sigma-Aldrich), the titer of lentivirus was determined.

### RNA interference screening for autophagy-regulating genes

2 × 10^5^ K562 cells were transduced with above 16 pools of lentiviruses at a multiplicity of infection (MOI) of 2.5 using the standard spinning infection approach. The volume of viruses was calculated using the formula: (MOI × cell number)/titer. Infected cells were then selected with 0.5 μg/ml puromycin for 1 week to facilitate the insertion of shRNAs into the host cell genome. Cells were stained with 0.1 mM MDC (Sigma-Aldrich) for 10 minutes in dark at 37 °C. Cells with strong MDC fluorescence (top 1%) were collected using FACS (wavelength: excitation 335 nm and emission 525 nm). Genomic DNA was isolated immediately after FACS using the QIAmp DNA mini kit (QIAGEN). DNA fragments that contained shRNA sequences were amplified using PCR as previously described^76^. The primer set used for this reaction was (Forward: 5′-acgatacaaggctgttagagag-3′; Reverse: 5′-cgaaccgcaaggaaccttc-3′). The PCR products were the cloned into a TA vector (Thermo Fisher Scientific) and submitted for Sanger DNA sequencing. The sequencing primer was 5′-aaacccagggctgccttggaaaag-3′. shRNAs were identified by searching the database RNAi codex. To help identify as many candidates as possible, 500 PCR-amplified DNA fragments were sent for sequencing. Among 500 sequences, 336 different shRNAs were identified and more than 50 shRNAs were repeatedly sequenced at least one more time, suggesting that nearly all PCR-amplified shRNAs in autophagic K562 cells have been identified in our screen. Primary candidates were further validated using the Cyto-ID fluorescence spectrophotometric assay and LC3B immunoblotting. For experiments involving individual shRNAs, lentivirus was prepared as described above. K562 cells were transduced with viruses with NS shRNA or candidate shRNAs followed by 1-week puromycin (0.1–0.5 μg/ml) selection.

### Immunoblotting

Immunoblotting was performed as previously reported^34,77^. Antibodies used in this study include: anti-LC3B (Cell Signaling Technology®, 1:1000), anti-ACTB (β-actin) (Sigma-Aldrich, 1:5000) anti-KRAS, anti-LYN (Cell Signaling Technology®, 1:1000), anti-HCLS1 (Cell Signaling Technology®, 1:1000), anti-PLK1 (Cell Signaling Technology®, 1:1000), and anti-ETS2 (Santa Cruz Biotechnology, Inc., 1:200). Quantification of protein brand intensities was performed using Image J as described previously^34^. The band intensities of proteins of interest were first normalized to that of actin (ACTB), yielding relative intensities. The relative intensities of proteins of interest in candidate shRNA-treated cells were further normalized with that in NS shRNA-treated cells.

### Quantitative RT-PCR

mRNA levels of genes of interest were measured using qRT-PCR as described previously^76,78^. In brief, K562 cells treated with NS shRNA or individual candidate shRNAs were subject to total RNA isolation using Trizol (Thermo Fisher Scientific). 1 to 5 μg total RNA was used to generate cDNA using SuperScript III reverse transcriptase (Thermo Fisher Scientific). The mRNA levels of each candidate gene were determined using quantitative real-time PCR.

### Bioinformatic analyses

Potential protein-protein interaction was analyzed using the online program STRING v9.1 (http://string-db.org). Bait proteins included (1) proteins that directly participate in the autophagy process and (2) kinases that regulate autophagy. Results were exported as image files and modified using adobe illustrator. Lines between nodes represent potential interactions. Colors denote different assays. Gene ontology analyses were performed using the online program DAVID (https://david.ncifcrf.gov) or GeneCards database.

### Autophagy stage determination assay

Autophagy stage determination assay was previously described^34,35^. K562 cells that stably express NS shRNA or individual candidate shRNAs were treated with either vehicle (water or DMSO), chloroquine (2.5 μM for 6 hours), or bafilomycin A1 (5 nM for 48 hours). In some cases, these cells were treated with DMSO, PP242 (5 μM), or imatinib (1 μM) for 8–10 hours. The amount of autophagic compartments was measured using the Cyto-ID fluorescence spectrophotometric assay or LC3B immunoblotting as described above. The Cyto-ID level in cells treated with the combinations was compared to that of each treatment alone. The statistical difference was obtained using the Student’s *t* test.

## Electronic supplementary material

Supplemental data

## Data Availability

All data are available for sharing upon request.
