# Author Correction: A large-scale RNA interference screen identifies genes that regulate autophagy at different stages

**DOI:** 10.1038/s41598-019-42889-1

**Published:** 2019-06-10

**Authors:** Sujuan Guo, Kevin J. Pridham, Ching-Man Virbasius, Bin He, Liqing Zhang, Hanne Varmark, Michael R. Green, Zhi Sheng

**Affiliations:** 1 Virginia Tech Carilion Research Institute, Roanoke, VA 24016 United States; 2 Graduate Program in Translational Biology, Medicine, and Health, Virginia Tech, Blacksburg, VA 24061 United States; 3 Howard Hughes Medical Institute and Department of Molecular, Cell and Cancer Biology, University of Massachusetts Medical School, Worcester, MA 01605 United States; 4 Department of Computer Science, Virginia Tech, Blacksburg, VA 24061 United States; 5 The Novo Nordisk Foundation Center for Basic Metabolic Research, University of Copenhagen, Copenhagen, Denmark; 6 Department of Biological Sciences and Pathobiology, Virginia-Maryland College of Veterinary Medicine, Virginia Tech, Blacksburg, VA 24061 United States; 7 Department of Internal Medicine, Virginia Tech Carilion School of Medicine, Roanoke, VA 24016 United States; 8 Faculty of Health Science, Virginia Tech, Blacksburg, VA 24061 United States

Correction to: *Scientific Reports* 10.1038/s41598-018-21106-5, published online 12 February 2018

This original version of this Article contained typographical errors.

In the Abstract,

“Our RNA interference screen identifies identified genes that regulate autophagy at different stages, which helps decode autophagy regulation in cancer and offers novel avenues to develop autophagy-related therapies for cancer.”

now reads:

“Our RNA interference screen identified genes that regulate autophagy at different stages, which helps decode autophagy regulation in cancer and offers novel avenues to develop autophagy-related therapies for cancer.”

Additionally, in the Results section under the subheading ‘MDC stains autophagic K562 cells’,

“Immuno-detection of the lapidated lipidated form of LC3B (LC3B-II) located on the membrane of autophagosomes and GFP-LC3B fluorescence microscopy that monitors the formation of autophagosomes (GFP-LC3B puncta) are well-established autophagy assays^32,33^.”

now reads:

“Immuno-detection of the lipidated form of LC3B (LC3B-II) located on the membrane of autophagosomes and GFP-LC3B fluorescence microscopy that monitors the formation of autophagosomes (GFP-LC3B puncta) are well-established autophagy assays^32,33^.”

Finally, the Supplemental data file contained errors in Figures 5G and 5H where “Bafilomycin A1” was incorrectly labelled as “Bafilomucin A1”.

These errors have now been corrected in the HTML and PDF versions of the Article, and in the accompanying Supplemental data file.

