# Supplementary material for: A large-scale RNA interference screen identifies genes that regulate autophagy at different stages
Source: Sci Rep. 2018 Feb 12;8:2822. doi: 10.1038/s41598-018-21106-5 (PMC5809370; doi:10.1038/s41598-018-21106-5)

A large-scale RNA interference screen identifies genes that regulate autophagy at different stages

Sujuan Guo, Kevin J Pridham, Ching-Man Virbasius, Bin He, Liqing Zhang, Hanne Varmark, Michael R Green, and Zhi Sheng

Supplemental Materials

Table S1. Candidate validation using the Cyto-ID spectrophotometric assay.

Gene Symbol	Cyto-ID		Gene Symbol	Cyto-ID		Gene Symbol	Cyto-ID		Gene Symbol	Cyto-ID		Gene Symbol	Cyto-ID		Gene Symbol	Cyto-ID		Gene Symbol	Cyto-ID		Gene Symbol	Cyto-ID	
	MEAN	S.D.		MEAN	S.D.		MEAN	S.D.		MEAN	S.D.		MEAN	S.D.		MEAN	S.D.		MEAN	S.D.		MEAN	S.D.
**AACS**	**1.5**	0.0	C9orf72	1.2	0.2	EPO	0.8	0.1	HPS3	0.8	0.1	MARCH5	1.2	0.0	PIGS	0.7	0.1	SBNO1	1.3	0.2	**THTPA**	**1.4**	0.2
ABCC3	1.0	0.1	**CAMKV**	**1.5**	0.2	**ERGIC1**	**1.8**	0.1	**HSF2**	**1.7**	0.2	MBD5	1.0	0.1	PIK3CD	1.1	0.2	**SCD**	**1.4**	0.3	**TMEM27**	**1.6**	0.1
ABCD1	0.9	0.1	CBLL1	0.8	0.1	**ERLIN2**	**1.8**	0.1	HSPA1L	1.1	0.1	**MBTPS1**	**2.9**	0.6	**PIPOX**	**1.6**	0.1	SCGB1D1	1.3	0.1	TNFAIP2	1.2	0.2
**ACAT1**	**1.7**	0.2	**CBWD1**	**1.5**	0.2	**ERMP1**	**2.0**	0.0	HUWE1	1.0	0.2	**MESP2**	**1.9**	0.2	PLA2G2E	1.0	0.1	SCN3B	1.1	0.2	**TNNI3**	**1.9**	0.2
**ACTR3B**	**1.4**	0.1	**CCBP2**	**1.4**	0.3	**ERO1LB**	**1.4**	0.2	ICAM3	1.3	0.1	MMP27	0.9	0.0	**PLK1**	**1.6**	0.0	**SEPT1**	**1.6**	0.2	TOMM40	1.1	0.2
**ADAMTS2**	**1.4**	0.2	**CCDC108**	**1.7**	0.1	**ETS2**	**1.9**	0.5	**IDH3G**	**2.2**	0.1	**MOB3C**	**1.8**	0.2	PLK4	1.0	0.2	**SERP1**	**2.0**	0.1	TRH	1.1	0.1
ADAMTS6	1.1	0.1	**CCDC19**	**1.5**	0.1	EYA3	0.7	0.1	IFT74	1.1	0.2	MOCS2	0.9	0.1	PLP1	1.1	0.0	SERPINB7	1.0	0.0	**TRPV6**	**1.7**	0.0
**AGPS**	**1.5**	0.3	**CCDC77**	**1.5**	0.1	**FABP6**	**1.4**	0.2	**IGF2R**	**1.8**	0.4	MORG1	1.1	0.2	PODN	0.8	0.0	**SERPINB9**	**1.5**	0.1	TSPAN9	1.0	0.1
**AK9**	**1.5**	0.1	CCL4	1.1	0.2	FAM120A	0.7	0.1	IGFBP3	0.8	0.0	MPG	1.3	0.1	**POLR3D**	**3.1**	0.3	**SERPINF2**	**1.6**	0.2	TXNDC16	1.0	0.1
**AKIP1**	**1.6**	0.3	**CCR9**	**1.4**	0.1	**FAM13C1**	**1.7**	0.1	**IGSF1**	**2.5**	0.1	**MTHFD2L**	**1.4**	0.2	PPFIA4	1.1	0.2	SET	1.0	0.2	TYROBP	0.9	0.1
**AKR1C3**	**1.4**	0.1	CD276	0.7	0.1	FAM167A	0.6	0.0	IL1F5	1.2	0.3	MUC7	1.3	0.1	**PPP3R2**	**2.3**	0.3	SFRS2B	0.8	0.1	**U2SURP**	**1.8**	0.1
**ALDOC**	**1.5**	0.2	CD38	1.0	0.1	FARP2	1.1	0.2	**IL2**	**1.4**	0.1	MYCBPAP	1.2	0.3	PRCP	1.1	0.2	SGK493	1.1	0.2	UBA6	1.3	0.2
**ALG10**	**1.8**	0.2	**CD8B**	**1.4**	0.2	FAS	0.9	0.1	INHBB	1.1	0.2	**MYOF**	**1.6**	0.2	**PRKCD**	**2.4**	0.3	SGPL1	0.9	0.1	UBE2D3	1.0	0.1
AMMECR1	1.2	0.1	CDKL3	1.1	0.2	FBN1	0.8	0.1	INPP5B	0.8	0.1	NCOA2	1.3	0.5	**PRKG1**	**1.4**	0.2	SGTB	1.1	0.1	UCP2	1.3	0.2
AOX1	1.1	0.1	CDKL4	1.3	0.1	**FBXO38**	**2.3**	0.2	**IPMK**	**1.4**	0.0	**NCS1**	**1.9**	0.2	PSG4	1.1	0.1	SH3BP5L	1.3	0.1	UCP3	0.8	0.0
AP3B1	1.3	0.2	CEACAM3	0.8	0.1	FBXO44	1.0	0.2	ITIH2	1.2	0.1	NEFL	1.1	0.3	PTCH1	1.2	0.2	SH3RF1	1.0	0.1	**UNC5CL**	**2.0**	0.3
AQP12A	0.9	0.1	**CENPH**	**1.6**	0.2	**FGL2**	**1.5**	0.3	**ITIH5L**	**1.8**	0.0	**NIPA2**	**2.0**	0.2	**PTDSS1**	**3.7**	0.3	SLC10A3	1.1	0.1	UNQ9370	0.7	0.0
ARL11	0.9	0.1	CFL1	0.9	0.1	**FLJ32214**	**1.8**	0.2	**JAK1**	**1.4**	0.1	NKX2-8	0.8	0.3	**PTGER3**	**1.8**	0.2	**SLC25A18**	**2.4**	0.1	**USP9X**	**1.5**	0.2
**ATF5***	**1.5**	0.0	CHRM5	1.3	0.4	FLVCR1	1.2	0.1	**JARID1C**	**1.8**	0.3	NME1	1.3	0.0	RAB4A,	1.1	0.1	SLC25A27	1.0	0.1	**UTP15**	**1.5**	0.2
ATF5*	0.9	0.1	CLEC2A	1.3	0.1	FMR1	0.7	0.1	JTV1	1.0	0.2	NRTN	1.0	0.1	RABGGTA	1.0	0.1	**SLC25A33***	**1.8**	0.1	**VAMP7**	**2.0**	0.2
**ATOH7**	**1.8**	0.4	**CLECL1**	**1.5**	0.2	FOLR2	1.0	0.1	**KCNG4**	**2.2**	0.2	**NSDHL**	**1.7**	0.1	**RAD21**	**1.9**	0.2	SLC25A33*	1.1	0.1	**VGLL3**	**2.5**	0.5
ATP10D	1.0	0.1	**CLTA**	**1.4**	0.1	FOXI2	0.7	0.0	KCNQ5	0.9	0.1	NTNG1	0.7	0.1	RAG2	1.2	0.2	**SLC2A8**	**2.3**	0.3	VWF	1.2	0.1
AZIN1	1.2	0.2	**CNOT2**	**2.5**	0.1	FRMD5	1.2	0.1	**KCNS1**	**1.5**	0.2	NUDT7	1.0	0.0	RBBP5	0.9	0.1	**SLC6A11**	**2.1**	0.6	**WBP1L***	**2.1**	0.2
**B3GALT1**	**2.2**	0.1	CNTNAP5	0.9	0.0	**FTSJ1**	**1.7**	0.0	KIAA0494	1.2	0.1	OPLAH*	1.0	0.1	REEP4	1.2	0.2	**SLC6A12**	**1.7**	0.5	**WBP1L***	**1.4**	0.1
**BCL2L10**	**2.0**	0.2	**COX11**	**1.5**	0.1	**GALT**	**1.7**	0.2	KIAA1958	1.0	0.1	OPLAH*	1.0	0.1	**RHCE**	**1.5**	0.3	SNAP29	1.3	0.1	**WDR92**	**2.8**	0.1
BIVM	1.0	0.1	**CPXM1**	**1.5**	0.1	**GAPT**	**1.9**	0.2	**KRAS***	**1.5**	0.2	**OR2T10**	**2.0**	0.0	RHCG	1.3	0.1	SNAPC1	1.0	0.1	WNT8B	0.7	0.0
BMI1	1.1	0.2	CROT	1.2	0.2	GAS2L1	1.2	0.4	**KRAS***	**1.4**	0.2	**OR51D1**	**1.5**	0.1	**RNASEH1**	**1.7**	0.2	SPTY2D1	0.6	0.0	**YWHAZ**	**1.4**	0.1
BMP2	1.3	0.1	CTRB1	1.0	0.4	GBF1	0.6	0.0	**LINC00467**	**1.5**	0.1	OR52M1	1.3	0.1	**RNF144A**	**1.8**	0.2	**SRMS**	**1.4**	0.4	**ZC3H11A**	**1.5**	0.1
**BMPER**	**2.3**	0.3	**CUBN**	**2.5**	0.2	**GGCT**	**1.5**	0.4	**LIPE**	**1.5**	0.1	OR5P3	0.7	0.0	**RNF175**	**1.5**	0.3	ST8SIA1	1.0	0.0	ZCCHC12	1.2	0.0
BRF1	1.2	0.2	CXCL10	1.2	0.0	**GH2**	**1.6**	0.5	**LOC100131384**	**1.8**	0.1	OR8B8	1.3	0.0	ROR2	1.1	0.2	STAC	1.0	0.1	**ZFP1**	**1.6**	0.0
**BVES**	**1.5**	0.1	**DCX**	**1.9**	0.1	GJB5	1.3	0.2	LOC123688	1.1	0.2	**PAH***	**2.4**	0.1	**RP9**	**2.4**	0.3	STX5	1.0	0.1	ZNF134	1.0	0.1
**C12orf4**	**1.9**	0.3	DDX39*	0.9	0.1	**GLO1**	**1.7**	0.3	LOC203547	0.8	0.1	**PAH***	**1.9**	0.2	**RPL29**	**1.4**	0.2	SYTL4	0.9	0.0	ZNF154	1.0	0.1
C12orf60	1.1	0.1	DDX39*	0.8	0.1	**GPR15**	**1.5**	0.3	LOC341112	1.1	0.1	**PARVA**	**1.7**	0.1	RPP38	1.2	0.1	**T**	**3.2**	0.7	**ZNF197**	**2.3**	0.0
**C1QL3**	**1.4**	0.1	DDX58	1.0	0.1	**GPR19**	**1.4**	0.2	LOC388503	1.1	0.2	PCDH7	1.2	0.2	RPS6KB1	0.9	0.1	TAF1C	0.8	0.0	ZNF219	0.9	0.1
C21orf7	1.1	0.1	DHX36	0.6	0.1	GPS2	1.2	0.1	LOC729516	0.8	0.1	PCDHA12	1.2	0.2	RRAGD	1.1	0.1	TBX15	1.6	0.3	**ZNF330**	**1.5**	0.1
C22orf23	0.8	0.0	DIMT1L	1.0	0.1	**GRHL1**	**1.8**	0.3	LPA	1.0	0.2	**PCDHB7**	**1.5**	0.2	RSHL3	1.0	0.1	**TCEAL1**	**1.5**	0.1	ZNF434	1.2	0.2
C2orf67	1.7	0.2	**DIRC1**	**2.0**	0.2	**GRK4**	**2.0**	0.2	**LPCAT2**	**2.1**	0.3	PDAP1	1.1	0.1	RSPH1	1.0	0.3	**TCERG1**	**2.1**	0.3	**ZNF521**	**3.8**	0.3
C5orf25	1.2	0.2	DNA2	1.1	0.1	**H6PD**	**1.5**	0.1	LPPR4	0.6	0.1	**PEX2**	**2.3**	0.1	**RSPO4**	**1.5**	0.4	TCHHL1	0.8	0.1	**ZNF540**	**1.6**	0.1
**C6orf118**	**1.5**	0.2	**DNASE1**	**1.9**	0.2	**HCLS1**	**1.6**	0.0	LRP6	0.9	0.1	PFDN4	1.2	0.2	**S100A4**	**2.1**	0.2	**TCP11**	**1.4**	0.0	**ZNF568**	**1.5**	0.2
C6orf123	1.0	0.1	**DNASE1L1**	**2.0**	0.1	HDDC2	1.3	0.0	LUZP1	1.3	0.1	**PGLYRP3**	**1.9**	0.1	S100Z	1.3	0.2	TCTE1	1.1	0.0	ZNF600	1.1	0.2
C6orf190	0.4	0.0	EFCAB3	1.3	0.3	HIAT1	1.3	0.0	**LVRN**	**1.4**	0.0	**PHACTR1**	**2.2**	0.1	SAMD7	1.3	0.1	THAP2	1.6	0.2	**ZNF639**	**1.5**	0.1
C6orf192	0.9	0.1	EPN1	1.3	0.2	HMGB1	0.9	0.2	**LYN**	**1.5**	0.4	**PHF14**	**1.4**	0.3	**SASH3**	**1.7**	0.1	**THBS4**	**2.0**	0.1	**ZNF663**	**1.4**	0.1

Note: The Cyto-ID fluorescence of each candidate gene shRNA was normalized to that of the control nonsilencing (NS) shRNA (set as 1.0). Candidate genes with ≥ 1.4-fold increase were highlighted in bold. *Genes that were targeted by multiple shRNAs. S.D. refers to standard deviation from three independent experiments.

Table S2. Candidate validation using the LC3B immunoblotting assay.

Gene Symbol	LC3B-II/ACTB	Gene Symbol	LC3B-II/ACTB	Gene Symbol	LC3B-II/ACTB	Gene Symbol	LC3B-II/ACTB	Gene Symbol	LC3B-II/ACTB	Gene Symbol	LC3B-II/ACTB	Gene Symbol	LC3B-II/ACTB	Gene Symbol	LC3B-II/ACTB
AACS	**2.9**	*C6orf118*	0.9	***ERGIC1***	**2.7**	***GRK4***	**4.0**	***LVRN***	**1.6**	***PIPOX***	**4.0**	*SERPINB9*	1.3	***UBA6***	**1.7**
ACAT1	**2.2**	***CAMKV***	**3.3**	***ERLIN2***	**4.9**	*H6PD*	0.9	***LYN***	**3.5**	***PLK1***	**1.4**	*SERPINF2*	0.7	***UNC5CL***	**4.0**
ACTR3B	1.2	***CBWD1***	**2.7**	*ERMP1*	1.2	***HCLS1***	**4.0**	***MBTPS1***	**1.9**	***POLR3D***	**1.7**	***SLC25A18***	**3.6**	***USP9X***	**1.9**
ADAMTS2	**1.8**	*CCBP2*	1.3	*ERO1LB*	0.9	***HSF2***	**3.3**	***MESP2***	**2.4**	***PPP3R2***	**2.5**	***SLC25A33***	**2.1**	***UTP15***	**3.0**
AGPS	**4.3**	***CCDC108***	**4.9**	***ETS2***	**3.1**	*IDH3G*	1.2	***MOB3C***	**4.1**	***PRKCD***	**2.3**	***SLC2A8***	**3.7**	***VAMP7***	**3.6**
AK9	**4.0**	***CCDC19***	**2.2**	***FABP6***	**3.7**	***IGF2R***	**3.0**	***MTHFD2L***	**5.2**	***PTDSS1***	**1.9**	***SLC6A11***	**2.4**	***VGLL3***	**4.8**
AKIP1	**2.8**	***CCDC77***	**2.3**	***FAM13C1***	**1.9**	***IGSF1***	**3.3**	***NCS1***	**1.4**	***PTGER3***	**1.8**	***SLC6A12***	**4.0**	***WBP1L***	**5.2**
AKR1C3	**2.6**	***CD8B***	**2.4**	***FBXO38***	**5.0**	***IL2***	**3.4**	*NIPA2*	0.6	***RAD21***	**1.5**	*T*	1.3	***WBP1L***	**2.6**
ALDOC	1.2	***CENPH***	**1.6**	*FGL2*	0.7	*ITIH5L*	1.0	***NSDHL***	**3.5**	*RHCE*	1.1	***TBX15***	**2.4**	***WDR92***	**3.5**
ALG10	1.2	***CLECL1***	**1.4**	***FLJ32214***	**1.8**	***JAK1***	**1.4**	***OR2T10***	**1.3**	***RNASEH1***	**1.5**	***TCEAL1***	**1.5**	***YWHAZ***	**1.9**
ATF5	**6.6**	***CLTA***	**1.5**	*FTSJ1*	0.7	***JARID1C***	**1.4**	***OR51D1***	**1.8**	*RNF175*	1.1	***TCERG1***	**2.8**	***ZC3H11A***	**1.6**
ATOH7	0.9	***CNOT2***	**1.4**	***GALT***	**3.1**	***KCNG4***	**2.8**	***PAH***	**2.0**	***RP9***	**3.2**	***TCP11***	**3.5**	***ZFP1***	**4.0**
B3GALT1	**2.9**	***COX11***	**3.4**	***GAPT***	**4.6**	***KCNS1***	**2.1**	***PAH***	**1.8**	*RPL29*	1.2	***THAP2***	**2.1**	***ZNF197***	**2.9**
BCL2L10	**1.9**	***CPXM1***	**1.8**	***GGCT***	**1.4**	***KRAS***	**5.5**	***PARVA***	**3.3**	***RSPO4***	**2.2**	***THBS4***	**4.9**	***ZNF330***	**1.9**
BMPER	**2.1**	***CUBN***	**1.7**	***GH2***	**3.6**	*KRAS*	1.1	*PCDHB7*	1.0	***S100A4***	**3.5**	***THTPA***	**2.7**	*ZNF521*	1.0
BVES	**3.3**	***DCX***	**1.8**	***GLO1***	**1.7**	***LINC00467***	**1.4**	***PEX2***	**1.6**	***SASH3***	**1.9**	*TMEM27*	1.2	***ZNF540***	**3.7**
C12orf4	1.3	***DIRC1***	**4.9**	*GPR15*	1.3	***LIPE***	**2.2**	***PGLYRP3***	**2.4**	***SCD***	**4.5**	***TNNI3***	**1.4**	***ZNF568***	**3.6**
C1QL3	**3.2**	***DNASE1***	**1.4**	***GPR19***	**1.4**	***LOC100131384***	**1.6**	***PHACTR1***	**1.4**	***SEPT1***	**3.7**	***TRPV6***	**4.1**	***ZNF639***	**3.8**
C2orf67	0.6	***DNASE1L***	**2.4**	***GRHL1***	**1.6**	***LPCAT2***	**1.4**	***PHF14***	**1.6**	***SERP1***	**1.5**	***U2SURP***	**3.2**	***ZNF663***	**3.9**

Note: The LC3B-II/ACTB intensity of each candidate gene shRNA was normalized to that of the control NS shRNA (set as 1.0). Candidate genes with ≥ 1.4-fold increase were highlighted in bold.

Table S3. Quantitative RT-PCR to determine knockdown efficiency.

Gene symbol	Candidates/NS		Gene symbol	Candidates/NS		Gene symbol	Candidates/NS		Gene symbol	Candidates/NS		Gene symbol	Candidates/NS		Gene symbol	Candidates/NS	
	Average	Standard Deviation		Average	Standard Deviation		Average	Standard Deviation		Average	Standard Deviation		Average	Standard Deviation		Average	Standard Deviation
AACS	Not detected		CLTA	0.14	0.01	GPR19	0.13	0.05	MOB3C	0.50	0.00	RP9	0.34	0.01	U2SURP	0.48	0.00
ACAT1	0.05	0.00	CNOT2	0.46	0.02	GRHL1	0.24	0.00	MTHFD2L	0.80	0.06	RSPO4	Not detected		UBA6	0.26	0.02
ADAMTS2	0.33	0.10	COX11	0.10	0.01	GRK4	0.25	0.13	NCS1	0.11	0.01	S100A4	0.22	0.02	UNC5CL	0.58	0.09
AGPS	0.48	0.09	CPXM1	0.08	0.02	HCLS1	0.43	0.04	NSDHL	0.50	0.01	SASH3	0.35	0.09	USP9X	0.85	0.04
AK9	0.29	0.16	CUBN	0.35	0.14	HSF2	0.48	0.01	OR51D1	Not detected		SCD	0.16	0.01	UTP15	0.39	0.01
AKIP1	0.12	0.01	DCX	0.68	0.18	IGF2R	0.37	0.02	PAH	0.43	0.49	SEPT1	0.30	0.16	VAMP7	0.12	0.01
AKR1C3	0.39	0.06	DIRC1	0.17	0.03	IGSF1	0.31	0.01	PAH	1.09	0.28	SERP1	0.19	0.01	VGLL3	0.37	0.14
ATF5	0.20	0.01	DNASE1	1.41	0.06	IL2	0.89	0.12	PARVA	1.48	0.04	SLC25A18	1.01	0.11	WBP1L	0.34	0.04
B3GALT1	1.20	0.05	DNASE1L1	0.19	0.02	JAK1	0.22	0.01	PEX2	0.45	0.02	SLC25A33	0.15	0.00	WBP1L	0.06	0.00
BCL2L10	1.38	0.48	ERGIC1	0.18	0.01	JARID1C	0.62	0.00	PGLYRP3	0.31	0.06	SLC2A8	0.30	0.02	WDR92	1.03	0.05
BMPER	Not detected		ERLIN2	0.36	0.12	KCNG4	0.70	0.06	PHACTR1	Not detected		SLC6A11	Not detected		YWHAZ	0.04	0.00
BVES	0.27	0.05	ETS2	0.45	0.07	KCNS1	Not detected		PHF14	0.33	0.01	SLC6A12	1.16	0.02	ZC3H11A	1.15	0.02
C1QL3	0.46	0.04	FABP6	0.17	0.07	KRAS	0.44	0.02	PIPOX	0.23	0.11	TBX15	0.43	0.17	ZFP1	0.20	0.04
CAMKV	0.13	0.01	FAM13C1	0.40	0.28	LINC00467	0.35	0.02	PLK1	0.37	0.01	TCEAL1	1.44	0.02	ZNF197	0.49	0.03
CBWD1	0.17	0.00	FBXO38	0.18	0.04	LIPE	1.15	0.41	POLR3D	0.42	0.03	TCERG1	0.29	0.02	ZNF330	0.39	0.03
CCDC108	0.15	0.01	FLJ32214	0.59	0.19	LOC100131384	1.36	0.06	PPP3R2	0.30	0.05	TCP11	0.72	0.12	ZNF540	0.71	0.04
CCDC19	0.89	0.14	GALT	0.68	0.18	LPCAT2	0.31	0.04	PRKCD	0.17	0.01	THAP2	0.42	0.01	ZNF568	1.02	0.47
CCDC77	0.13	0.00	GAPT	1.62	0.36	LVRN	1.09	0.06	PTDSS1	0.04	0.00	THBS4	0.92	0.31	ZNF639	0.16	0.03
CD8B	Not detected		GGCT	0.05	0.00	LYN	0.12	0.00	PTGER3	0.54	0.29	THTPA	0.72	0.02	ZNF663	1.09	0.18
CENPH	0.02	0.01	GH2	0.31	0.00	MBTPS1	0.50	0.04	RAD21	0.90	0.05	TNNI3	0.08	0.00			
CLECL1	0.28	0.16	GLO1	0.13	0.00	MESP2	0.48	0.05	RNASEH1	0.30	0.01	TRPV6	0.81	0.19			

Note: The mRNA levels of each gene in cells receiving an shRNA of this gene were normalized to that of cells treated with the NS shRNA (set as 1.0). Genes with ≤ 0.5 reduction of mRNA levels were highlighted in orange.

Table S4. Capability of ARGs to induce the formation of autophagic compartments.

Ability to induce the formation of autophagic compartments	Autophagy-regulating genes
High (14)	*DIRC1, DNASE1L1, FBXO38, IGSF1, PAH, PPP3R2, PRKCD, RP9, SLC2A8, TCERG1, VAMP7, VGLL3, WBP1L, ZNF197*
Medium (46)	*ACAT1, AGPS, AK9, AKIP1, AKR1C3, ATF5, BVES, C1QL3, CAMKV, CBWD1, CCDC108, CCDC77, CNOT2, COX11, CUBN, ERGIC1, ERLIN2, ETS2, FABP6, GH2, GRK4, HCLS1, HSF2, IGF2R, KRAS, LPCAT2, LYN, MBTPS1, MESP2, MOB3C, NSDHL, PGLYRP3, PIPOX, POLR3D, PTDSS1, S100A4, SCD, SEPT1, SERP1, SLC25A33, TBX15, THAP2, U2SURP, UTP15, ZFP1, ZNF639*
Low (22)	*ADAMTS2, CENPH, CLECL1, CLTA, CPXM1, FAM13C1, GGCT, GLO1, GPR19, GRHL1, JAK1, LINC00467, NCS1, PEX3, PHF14, PLK1, RNASEH1, SASH3, TNNI3, UBA6, YWHAZ, ZNF330*

Note: Based on the results from Cyto-ID fluorescence spectrophotometric assay (Fig. 3B and Table S1) and LC3B immunoblotting (Fig. 3C-D and Table S2), the ability of ARG shRNAs to induce the formation of autophagic compartments was defined as: (1) High, if there was a ≥ 2-fold increase in both Cyto-ID and LC3B-II; (2) Medium, if there was a ≥ 2-fold increase of either Cyto-ID or LC3B-II; (3) Low, if there was a 1.4- to 1.9-fold increase of Cyto-ID and/or LC3B-II.

Table S5. Autophagy stage analysis in K562 cells treated with ARG shRNAs and/or chloroquine.

Gene Symbols	shRNA		Chloroquine		Gene Symbols	shRNA		Choloquine		Gene Symbols	shRNA		Chloroquine	
	Mean	SD	Mean	SD		Mean	SD	Mean	SD		Mean	SD	Mean	SD
NS1	1.000619	0.182031	1.934027	0.112883	NS5	1.000458	0.069730	2.047740	0.253824	NS9	0.999668	0.009547	1.594410	0.080959
CENPH	1.595201	0.020595	2.485301	0.206761	ACAT1	1.392064	0.001333	2.716857	0.167044	AGPS	1.497519	0.049561	2.438228	0.407913
**FAM13C1**	1.469508	0.046942	1.924864	0.166314	CPXM1	1.808584	0.058201	2.804015	0.188532	ETS2	3.354852	0.206075	4.444095	0.123183
JAK1	1.744462	0.021139	2.724005	0.290611	**GRK4**	1.969299	0.247016	2.570551	0.122606	**MOB3C**	1.475777	0.243866	1.811258	0.134677
MBTPS1	1.802524	0.010501	2.948791	0.351960	**HSF2**	1.571294	0.120211	2.498185	0.315328	PIPOX	1.694300	0.346489	2.636014	0.344167
**PLK1**	1.576458	0.258160	2.382783	0.032332	**PHF14**	1.448262	0.152594	2.361625	0.354707	S100A4	1.637692	0.093221	1.955229	0.066382
PPP3R2	2.119571	0.117374	2.829679	0.413709	**PTDSS1**	2.451556	0.114336	2.891095	0.365827	**U2SURP**	1.722377	0.025863	2.171680	0.309246
SCD	1.606597	0.186460	2.596972	0.241987	**TBX15**	1.374119	0.201438	2.180834	0.056474	**ZFP1**	1.963037	0.014031	2.109283	0.198114
TCERG1	1.626012	0.099890	2.410398	0.236991	**YWHAZ**	1.351682	0.177615	2.278872	0.148228	NS10	1.000216	0.047723	1.406934	0.132352
THAP2	1.761406	0.049527	2.733407	0.304303	NS6	0.999735	0.070226	2.115860	0.056685	**AKR1C3**	1.793041	0.297279	2.402697	0.190527
NS2	0.999470	0.054787	1.394594	0.242586	CNOT2	3.000301	0.052294	4.757733	0.601990	ATF5	1.474238	0.092203	2.038162	0.080895
ADAMTS2	1.970799	0.369494	2.992354	0.421247	**COX11**	1.349611	0.119103	2.218223	0.281095	**ERGIC1**	1.435520	0.209608	1.914072	0.165147
CUBN	1.829948	0.145021	2.573386	0.158900	GH2	2.144240	0.181689	3.246636	0.160367	FABP6	1.365169	0.129577	2.256143	0.274836
**DNASE1L1**	2.356814	0.650392	3.748881	0.403486	IGF2R	2.247165	0.159369	3.841260	0.277907	LINC00467	1.713080	0.125556	2.736193	0.201435
GRHL1	2.170174	0.070074	3.139624	0.339839	KRAS	2.127420	0.211115	3.472156	0.530725	UTP15	1.531804	0.078272	2.405968	0.374532
MESP2	1.922320	0.058684	2.482440	0.162714	LPCAT2	1.440216	0.031440	2.354927	0.116531	VAMP7	1.629825	0.029595	2.727679	0.243446
NCS1	1.464632	0.003620	2.560817	0.111782	NSDHL	1.386867	0.000233	2.245742	0.081834	NS11	0.999905	0.055335	1.398313	0.059188
PEX2	2.088296	0.010720	3.151712	0.271253	NS7	1.000109	0.234620	1.590380	0.068465	**SLC2A8**	1.543933	0.437153	2.156099	0.303874
POLR3D	2.282815	0.016437	3.086246	0.147483	AK9	1.809720	0.240336	3.531951	0.087394	NS12	0.996538	0.112554	1.638645	0.172291
**UBA6**	1.681870	0.202301	2.173606	0.131982	DIRC1	2.014401	0.100015	3.828415	0.659487	RP9	1.292372	0.209274	2.330396	0.127246
ZNF197	1.946509	0.170207	3.091169	0.289621	ERLIN2	1.690161	0.027217	3.219096	0.421454	SERP1	1.660197	0.117410	2.856202	0.212407
NS3	1.000604	0.006729	1.469603	0.056746	FBXO38	1.572248	0.180520	3.230834	0.279133	TNNI3	1.428521	0.111789	2.398735	0.211937
AKIP1	1.253413	0.108058	2.373596	0.163224	SEPT1	1.719317	0.279654	2.511857	0.259281	NS13	0.999512	0.042441	2.317069	0.246090
**CCDC77**	1.575151	0.327976	2.154585	0.101882	VGLL3	2.190006	0.391726	4.103983	0.542292	**C1QL3**	1.344979	0.008397	2.359021	0.252953
CLECL1	1.263422	0.094021	1.965180	0.140033	ZNF639	1.741143	0.001986	4.400130	0.060481	CLTA	1.960833	0.139688	3.509297	0.253615
PAH	1.737113	0.084048	2.472377	0.188858	**PRKCD**	1.156142	0.032779	1.874524	0.209342	**GGCT**	1.446936	0.148591	2.684141	0.318237
**PGLYRP3**	1.799751	0.294265	2.784234	0.086201	NS8	1.000761	0.016057	1.945498	0.521560	**RNASEH1**	1.213963	0.137206	2.282216	0.246090
**SASH3**	1.344719	0.244792	2.026481	0.092228	BVES	1.732428	0.233569	2.916644	0.370007	NS14	0.996325	0.143920	1.841456	0.100116
SLC25A33	2.280205	0.088323	3.005972	0.422296	**CBWD1**	1.364142	0.021605	2.362036	0.045838	LYN	1.315710	0.083637	2.123585	0.077302
WBP1L	1.432026	0.141873	1.992970	0.171829	CCDC108	2.011232	0.127540	3.099143	0.259411	NS15	0.999023	0.061969	1.370225	0.078858
ZNF330	1.575369	0.136054	2.183344	0.122330	**GLO1**	1.353329	0.004766	2.341212	0.251986	CAMKV	1.293277	0.124964	1.727192	0.022430
NS4	1.000814	0.086308	1.506172	0.063841	GPR19	2.014520	0.152623	3.667548	0.596187					
HCLS1	1.659466	0.578063	2.439711	0.430858	IGSF1	2.118822	0.245895	3.340908	0.177437					

Note: SD refers to standard deviation from three independent experiments.

Table S6. Autophagy stage analysis in K562 cells treated with ARG shRNAs and/or PP242.

Gene Symbols	shRNA		PP242		Gene Symbols	shRNA		PP242	
	Mean	SD	Mean	SD		Means	SD	Means	SD
NS1	1.000001	0.023815	2.085373	0.154344	*NS6*	1.000317	0.078042	2.020508	0.089766
GRK4	1.436322	0.225095	2.701204	0.254907	***COX11***	1.435158	0.136366	2.459485	0.139472
U2SURP	1.624655	0.148899	3.032930	0.282409	*ERGIC1*	1.610739	0.136340	2.044539	0.191020
YWHAZ	1.567508	0.120723	2.836741	0.210909	***GLO1***	1.676056	0.235481	2.344834	0.115829
ZFP1	1.656126	0.052042	2.787867	0.160684	***MOB3C***	1.562320	0.109312	2.794300	0.203166
NS2	1.000000	0.066895	1.714300	0.161000	*NS7*	1.000195	0.124722	2.376541	0.049441
DNASE1L1	1.435112	0.093860	2.553875	0.060506	***C1QL3***	1.476420	0.061718	4.633339	1.114527
PGLYRP3	1.252286	0.068504	2.382802	0.086661	***PLK1***	1.437949	0.062515	2.800026	0.262306
PTDSS1	1.222411	0.031572	2.385018	0.117197	***SLC2A8***	1.494294	0.058155	2.628240	0.229973
NS3	1.000002	0.090663	2.462786	0.018917	*NS8*	1.000203	0.040195	1.570568	0.098943
CBWD1	1.603848	0.113153	3.211210	0.056467	***CCDC77***	3.557308	0.662825	1.573777	0.276964
GGCT	1.632085	0.140487	3.258336	0.082211	***RNASEH1***	2.739408	0.193491	1.505716	0.022984
RNASEH1	1.470177	0.158778	3.225801	0.091767	***SASH3***	1.573777	0.276964	2.739408	0.193491
NS4	1.000002	0.138918	1.651325	0.179945	*NS9*	1.002266	0.021407	2.194668	0.159845
HSF2	1.273991	0.128565	2.036479	0.121379	***AKR1C3***	1.463435	0.119839	3.224797	0.647982
NS5	0.999408	0.074726	3.100808	0.176309	*NS10*	0.997990	0.103571	1.401542	0.193935
PHF14	1.549188	0.159635	2.891937	0.316077	***FAM13C1***	1.363106	0.113932	1.814193	0.107506
TBX15	1.568195	0.285548	2.707288	0.479760					
UBA6	1.431945	0.084370	3.280532	0.290283					

Note: SD refers to standard deviation from three independent experiments.

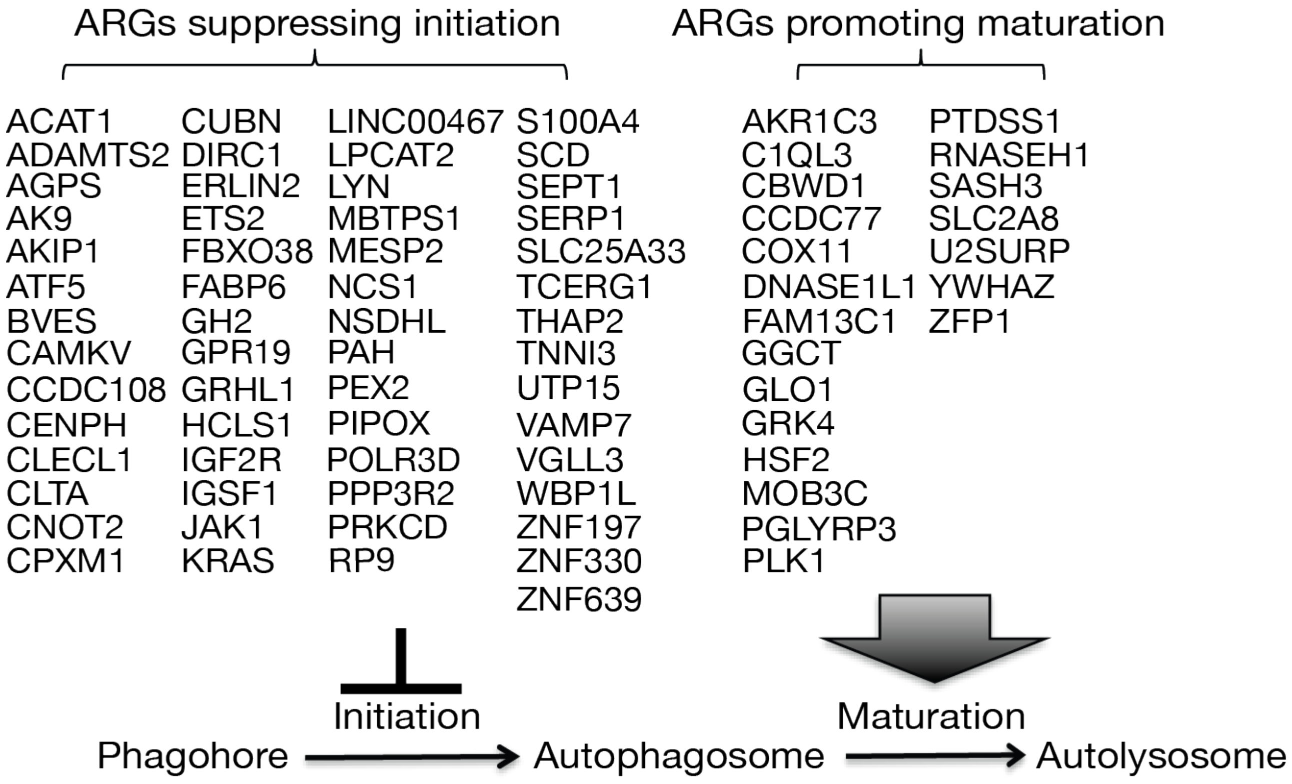

Fig. S1. ARGs regulate autophagy at different stages. The autophagy process includes two key steps: initiation and maturation. ARGs that suppress autophagy initiation and ARGs that promotes autophagy maturation are shown.

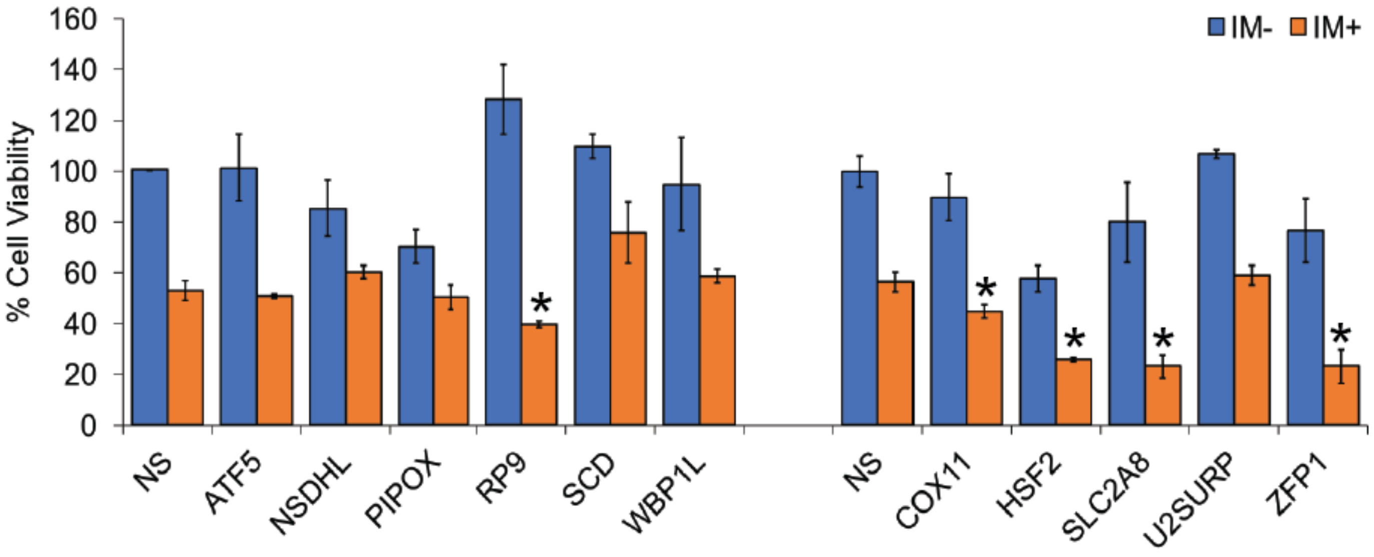

Fig. S2. Imatinib sensitivity in K562 cells upon depletion of ARGs. K562 cells were transduced with viruses of non-silencing (NS) shRNA or individual ARG shRNAs. Cells were then treated with DMSO (IM-) or 1 μM of imatinib (IM+) for 48 hours. Cell viability was monitored using the MTS cell viability assay. The error bar represents standard deviation from three independent experiments. Percentages of cell viability in K562 cells with ARG shRNAs were compared with the percentages of cell viability in K562 cells treated with NS shRNAs using the student *t* test. *$P < 0.05$.

Images of immunoblotting

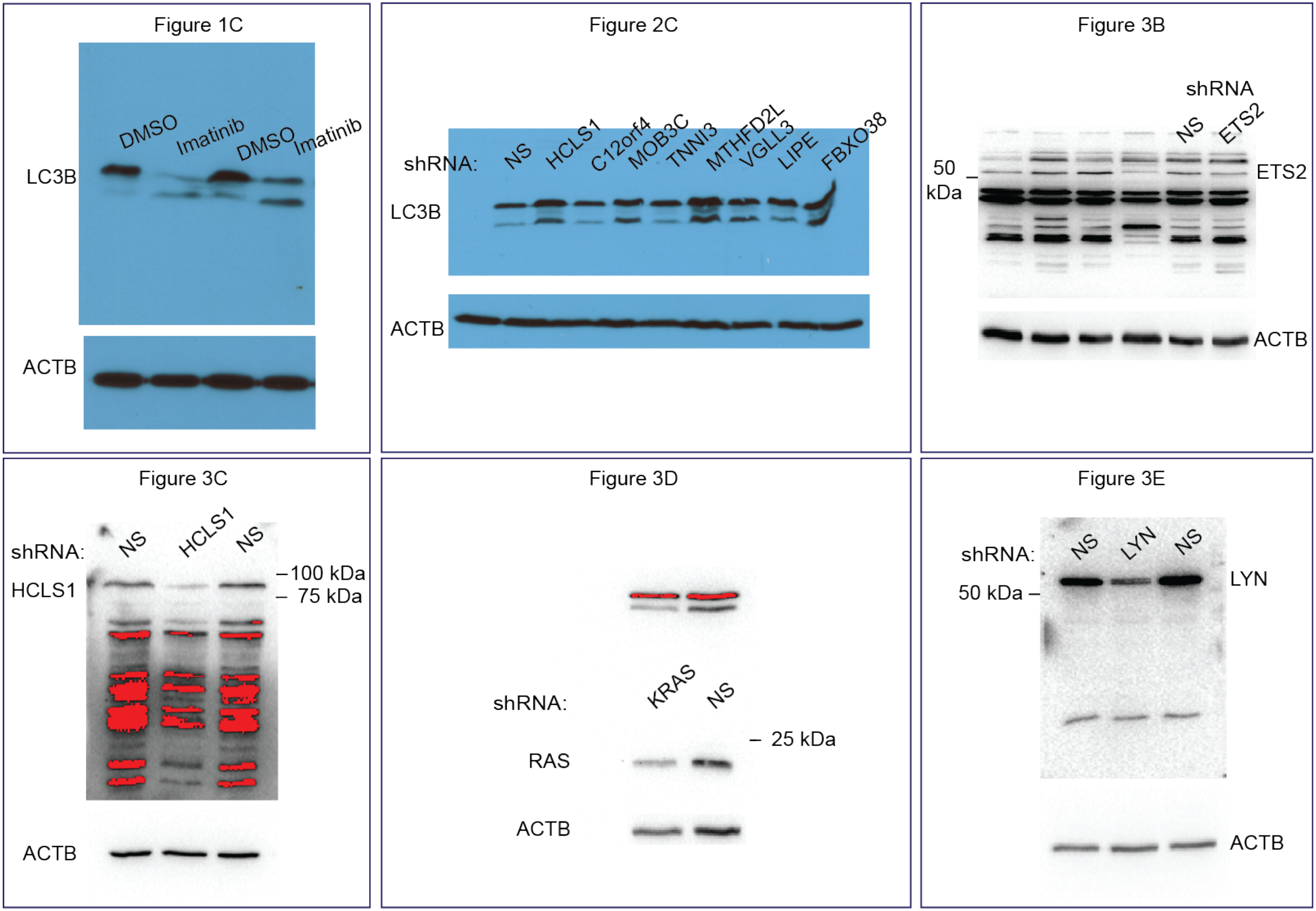

Figure 5B

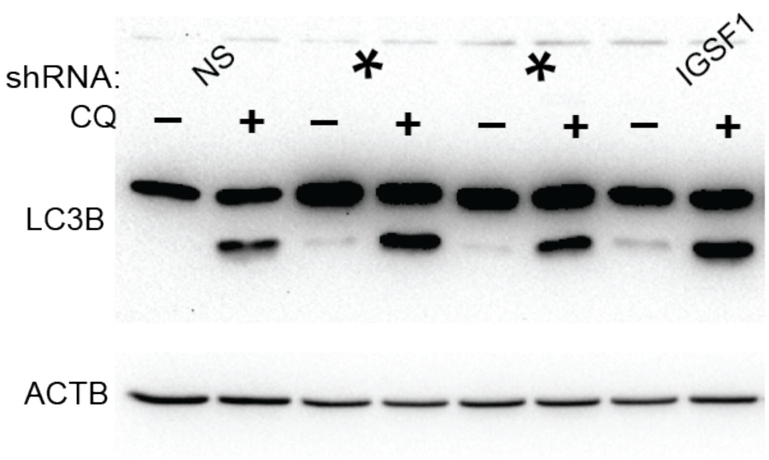

CQ: chloroquine. * not validated candidates in the primary screen.

Figure 5D

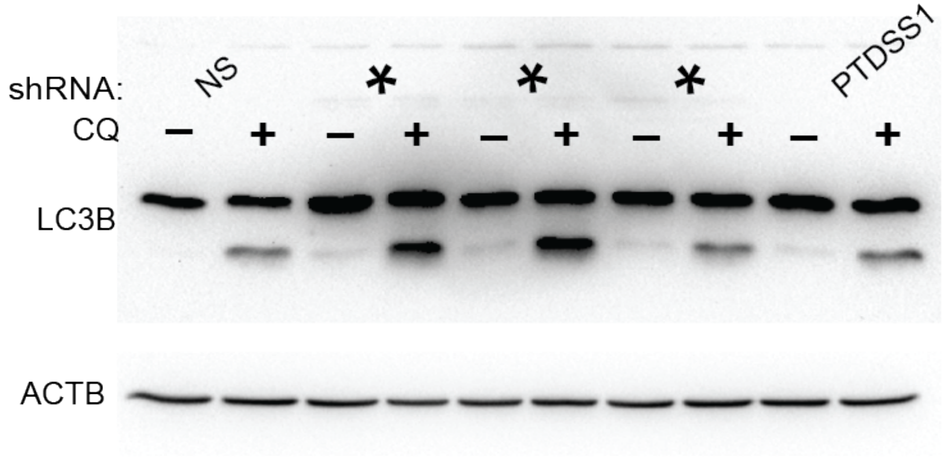

Figure 5E

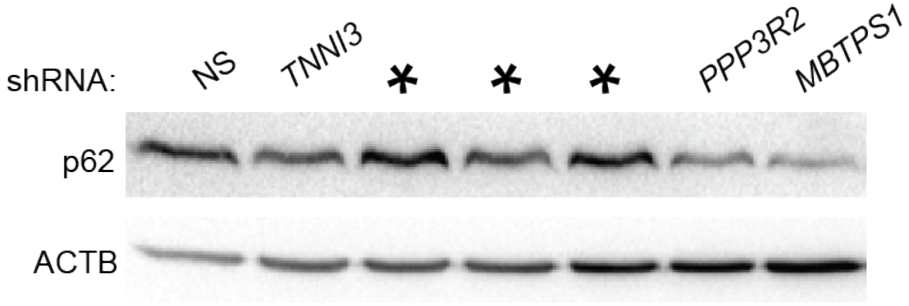

Figure 5F

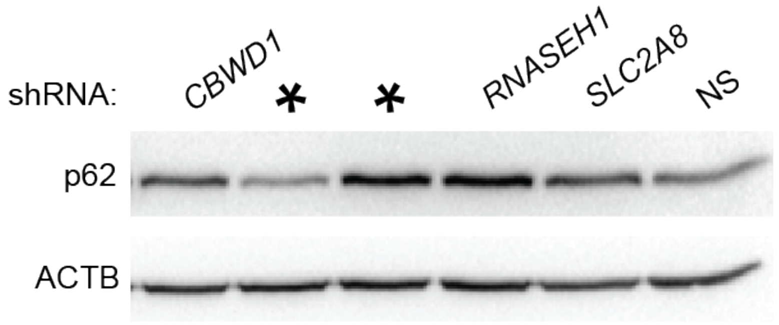

Figure 5G

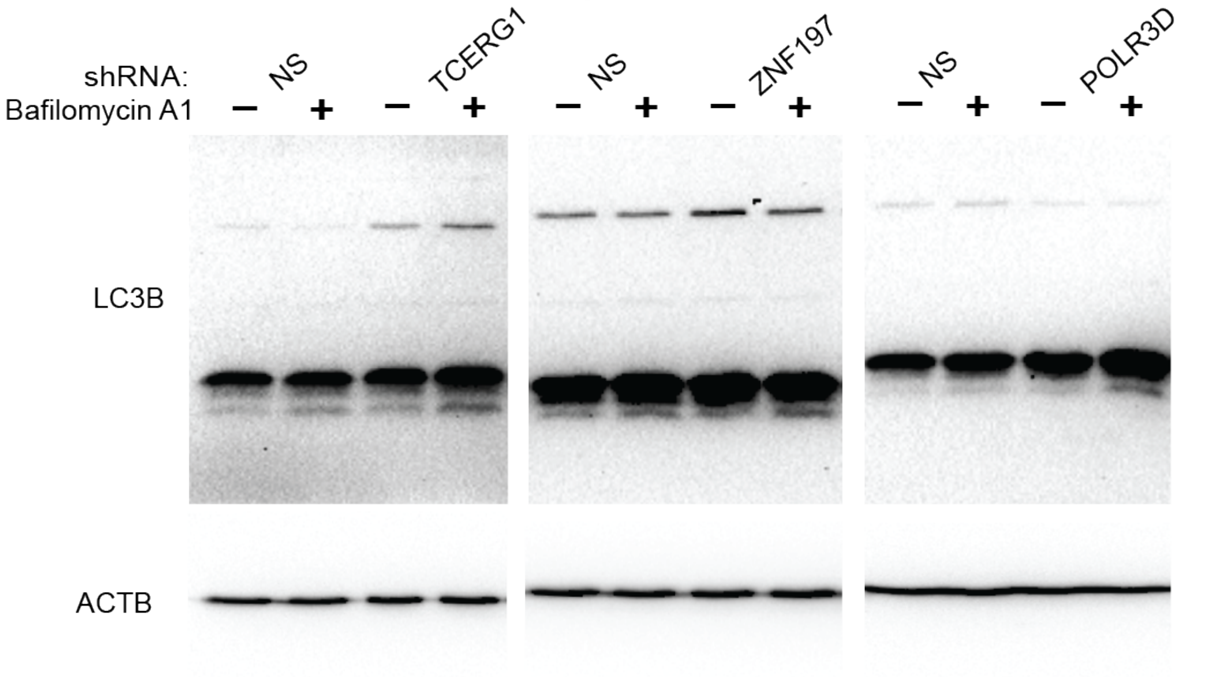

Figure 5H

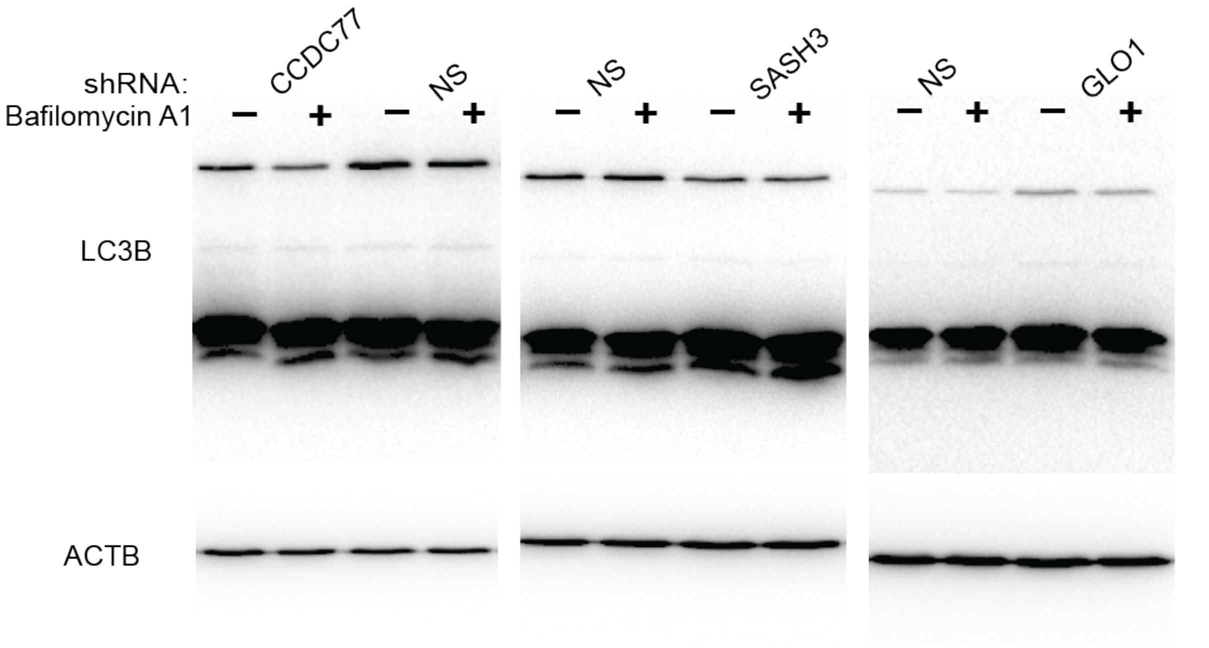

Supplement: Supplementary file 1 — Supplemental data [file 41598_2018_21106_MOESM1_ESM.pdf]
